# *Arabidopsis thaliana* phytochrome A sensory properties in canopy shade

**DOI:** 10.1073/pnas.2512201123

**Published:** 2026-01-27

**Authors:** Philip Prizeman-Green, Marissa Valdivia-Cabrera, Mengke Zhou, Ramon Grima, Karen J. Halliday

**Affiliations:** ^a^Institute of Molecular Plant Sciences, School of Biological Sciences, University of Edinburgh, Edinburgh EH9 3BF, United Kingdom; ^b^Centre for Engineering Biology, School of Biological Sciences, University of Edinburgh, Edinburgh EH9 3BF, United Kingdom

**Keywords:** light, phytochrome, *Arabidopsis*, photomorphogenesis, hypocotyl

## Abstract

Canopy shade environments can negatively impact plant growth and survival. Phytochrome A (phyA) is essential for seedling adaptation to deep shade, yet its role under moderate natural canopy conditions remains unclear. By expressing a phyA-nanoLUC reporter in *Arabidopsis thaliana*, we quantify diurnal fluctuations in phyA and its induction by low red to far-red (R:FR) ratio, simulating canopy shade. We uncover a key regulatory function for phyB, which increases phyA stability postdawn in unshaded conditions and modulates expression of *PHYA* in low R:FR. Further, we demonstrate that beyond its role as a deep shade sensor, phyA is an adept sensor of canopy shade, capable of detecting a range of R:FR ratios across light intensities. Mathematical modeling demonstrates that this property arises from the dynamic features of the phyA High Irradiance Response (HIR) mode of action. Interestingly, phyA synthesis is strongly induced by subtle reductions in R:FR ratio, and is robust to light perturbation, suggesting the phyA-sensory module is configured to detect modest shade. Spectral data from natural shade habitats provide ecological context for our laboratory findings. Physiological analysis indicates that under canopy shade phyA promotes seedling de-etiolation, organizes resource management, and accelerates reproductive development. We propose that this suite of responses, termed the “canopy adaptation strategy,” enhances survival chances under conditions where shade avoidance strategies are maladaptive.

## Significance

Adaptation to canopy shade is crucial for plant survival, yet understanding of this important response remains limited. This study highlights the crucial role of phytochrome A (phyA) in this survival strategy. Previous research established phyA as an important sensor for deep shade conditions. In our research, we demonstrate that phyA is highly sensitive to and can accurately detect even very mild shade. Additionally, contrary to initial assumptions, the distinctive characteristics of the phyA sensory module enable it to precisely gauge the intensity of shade. This advanced sensing ability allows plants to effectively conserve resources and accelerate their reproductive cycle when faced with persistent shade.

In natural ecosystems, vegetational shade presents a critical challenge for plants by reducing the light available for photosynthesis and detrimentally impacting growth. Remarkably, many plants are not only capable of detecting when they are in shade, but also differentiating between types of shade. Crucially, this ability to distinguish between shade environments produced, for example, by encroaching neighbors directly competing for available light and those cast by overstory canopies, enables plants to deploy adaptive growth strategies tailored to the specific scenario they face (1, 2).

At the heart of this shade detection system are the phytochrome photoreceptors. Phytochrome B (phyB) plays a pivotal role in eliciting the shade avoidance syndrome (SAS), which includes hypocotyl elongation in seedlings, as well as leaf hyponasty, petiole elongation, reduced leaf area, and accelerated flowering in adult plants (1, 3). By directing growth upward and outward, the SAS provides a competitive advantage to plants by improving leaf positioning and enhancing light interception among similarly sized neighbors (3–5). Conversely, adopting the SAS is considered to be a detrimental strategy in the most severe, “deep shade” environments (e.g., in thick undergrowth), where excessive elongation growth reduces survivability (6). Plants, therefore, utilize a second phytochrome in deep shade, phyA, to antagonize the SAS in developing seedlings (6–8). While the importance of phyA action in deep shade is well documented (6–8), the efficacy of phyA beneath milder vegetational canopies, produced either by tree cover or ground-level overtopping neighbors and frequently encountered in natural environments, remains uncertain. Interestingly, as with deep shade scenarios, the adaptive value of shade-induced elongation in some species, including rosette annuals like *Arabidopsis thaliana*, is believed to diminish beneath canopy cover (5, 9).

Light in canopy shade is both reduced in photosynthetically active radiation (PAR; 400 to 700 nm) and spectrally enriched with far-red (FR; 700 to 780 nm), relative to red (R; 600 to 700 nm), wavelengths (4, 10, 11). The degree of reduction in the R:FR ratio can be used to quantify the severity of foliar shade (11). Changes in the external prevalence of R and FR wavelengths are monitored by phytochromes, a dichromic class of photoreceptors that exist in photoreversible inactive (Pr) and active (Pfr) forms (4, 12). R light is absorbed by Pr (λ_max_ ~ 660 nm), which induces photoconversion to the active Pfr form. Conversely, FR absorption by Pfr (λ_max_ ~ 730 nm) reverses this process, shifting phytochrome molecules to the inactive, Pr state (12). While all phytochromes share these photosensory traits, their ways of operating differ markedly. Most notably, phyA and phyB, the two most prominent of the five phytochromes (phyA-E) in Arabidopsis, have divergent response behaviors (13–15).

Modeling approaches have been instrumental in creating a theoretical framework for understanding the operational properties of phyA and phyB (16–22). PhyA functions in either a Very Low Fluence Response (VLFR) mode, induced by weak irradiances of any wavelength, or a High Irradiance Response (HIR) mode, activated by continuous irradiation with FR (14). The phyA signaling module includes a photocycle-coupled, nuclear shuttling mechanism involving homologous carrier proteins FAR-RED ELONGATED HYPOCOTYL 1 (FHY1) and FHY1-LIKE (FHL) (17, 23, 24). This process, driven by photoconversion between the Pr and Pfr forms, involves several steps. Initially, phyA-Pfr in the cytoplasm binds to FHY1/FHL, promoting its transport into the nucleus. As the signaling capacity of phyA-Pfr is impaired in this complex, two further photoconversions are required within the nucleus, first to Pr, releasing FHY1/FHL, and then back to the biologically active Pfr form. Consequently, this recurring cycle facilitates the accumulation of phyA in the nucleus in environments rich in far-red light (17, 25).

In contrast, phyB operates in a Low Fluence Response (LFR) that is R/FR reversible. PhyB undergoes photoactivation in response to R light, resulting in translocation of phyB-Pfr from the cytoplasm to the nucleus to execute its signaling functions (16, 25). Absorption of FR irradiances, on the other hand, initiates the photoreversion of phyB-Pfr to -Pr, resulting in phyB nuclear expulsion (25). Model analysis has shown that the proportion of active (nuclear) phyB (Pfr/Ptot [Pr+Pfr]) under high irradiance light is strongly linked to the external R:FR (4, 22, 26, 27). This quality makes phyB effective in sensing reflected FR light from neighboring plants where light levels are typically high (11, 22). However, in lower irradiance scenarios, such as those encountered in a closed-canopy environment or, more fleetingly, as a result of cloudy weather, thermal reversion of Pfr back to Pr becomes increasingly influential on Pfr/Ptot (22). Consequently, phyB activity is believed to be more strongly impacted by temperature in such scenarios and less so by R:FR (22). Interestingly, in Arabidopsis accessions such as *Landsberg erecta, Wassilewskija,* or *Columbia* (studied here), no thermal reversion of phyA has been observed (28, 29). It is therefore worthwhile exploring the role of phyA in detecting canopy shade conditions where phyB may not serve as a reliable sensor of competition.

In this study, we set out to determine the operational range of phyA in canopy shade and how this relates to the unique properties of phyA HIR mode. To do this, we developed phyA-nLUC lines that provide quantitative readout of phyA abundance. Our data show phyA levels are highly dynamic, responding to even very subtle reductions in R:FR ratio indicative of mild canopy shade. Mathematical modeling and experimental analysis established that the specialized photosensory properties of phyA allow it to accurately detect changes in R:FR ratio. As a result, phyA serves as a sensitive detector of canopy shade, triggering an adaptive growth strategy for canopy adaptation.

## Results

### Quantitative Tracking of phyA Abundance Using phyA-nLUC Reporters.

**Fig. 1.**
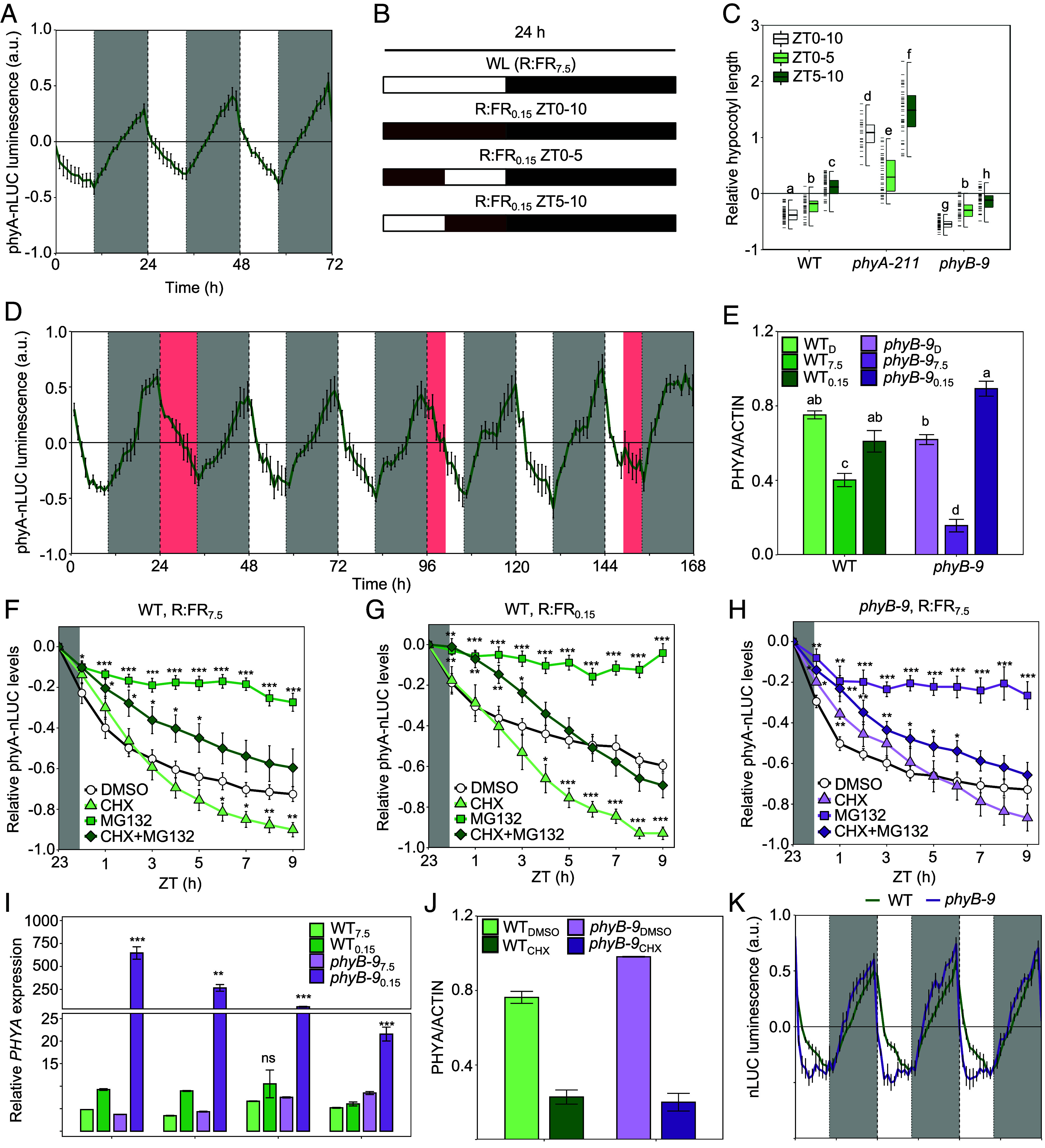
Low R:FR ratio light increases phyA abundance and activity at any time of day. (*A*) phyA-nLUC bioluminescence (arbitrary units, a.u.) in diurnal cycles (10L:14D) of white light (R:FR_7.5_). (*B*) Schematic of R:FR_0.15_ treatments (predicted Pfr/Ptot ~ 0.24) provided in *C* and *D*. (*C*) Relative hypocotyl lengths of 6-day-old WT, *phyA-211,* and *phyB-9* seedlings treated daily with R:FR_0.15_ from ZT0-10, ZT0-5, or ZT5-10. Relative length was calculated with respect to the length of each genotype in control conditions (R:FR_7.5_ [predicted Pfr/Ptot ~ 0.80]); i.e. relative change for each genotype = (length in R:FR_0.15_ treatment - average length in R:FR_7.5_)/average length in R:FR_7.5_. (*D*) PhyA-nLUC bioluminescence in seedlings treated with R:FR_0.15_ between 24 to 34 h, 96 to 101 h, or 149 to 154 h. (*E*) PhyA protein levels, quantified using a western blot and normalized to ACTIN, in a WT or *phyB-9* background just prior to dark–light transition (Darkness; D), or at midday (ZT5) in R:FR_7.5_ or R:FR_0.15_. Hourly abundance changes in phyA-nLUC expressed in WT (*F*–*H*) *phyB-9* seedlings treated with 50 μM MG132 and/or 200 μM CHX to block either proteasomal degradation, translation, or both; DMSO alone was used as a control. (*F*-*H*) Relative phyA-nLUC levels following the dark–light transition (ZT0) into a high (R:FR_7.5_; *F* and *H*) or low (R:FR_0.15_; *G*) day period, calculated with respect to the luminesce signal predawn (ZT23). Relative change [(value at ZTX – value at ZT23)/value at ZT23] calculated for each seedling, then averaged. (*I*) Abundance of *PHYA* transcripts (normalized to *PP2A* using the 2^−ΔΔCt^ method) in WT or *phyB-9* seedlings at 4 time points throughout R:FR_7.5_ or R:FR_0.15_ day periods using qRT-PCR. (*J*) PhyA protein levels (quantified western blot signal) in WT or *phyB-9* seedlings at ZT5 (R:FR_0.15_) when treated with CHX or a control (DMSO). (*E* and *J*) Anti-phyA antibody was used to detect native phyA, while anti-ACTIN served as an internal control. (*K*) PhyA-nLUC bioluminescence in 10L:14D cycles in WT (green line) or a *phyB-9* (purple line) background. (*A*, *D*, *F*–*H,* and *K*) Traces show mean signal produced by *n* ≥ 8 plants, measured at 1 h intervals; error bars show ± SEM. (*A*, *D,* and *K*) Background colors of each panel correspond to R:FR_7.5_ (white), R:FR_0.15_ (red), and night (gray) periods. (*C*) Box plots display: the median (*Center* line); *Upper* and *Lower* quartiles (box limits); 1.5× interquartile range (whiskers); individual data points (dashes on the left of boxes, *n* ≥ 25). (*E*, *I,* and *J*) Bars indicate the mean; error bars show ± SEM (*n* = 3). Letters denote statistically indistinguishable groups according to a Kruskal–Wallis test followed by a post hoc Dunn’s Test (Bonferroni correction) (*C*) or using an ANOVA followed by a Tukey’s HSD Test (*E*). (*F*–*J*) Asterisks indicate significant differences calculated using a Student’s *t* test at each time point from DMSO (*F*–*H* and *J*) or WT_7.5_ (*I*) controls (*P < 0.05; **P < 0.01; ***P < 0.001). All experiments were conducted in a background WL of 15 μmol m^−2^ s^−1^. Each experiment as repeated three times.

PhyA protein abundance is highly dynamic, exhibiting strong diel rhythmicity (30, 31). To quantitatively track this dynamic behavior, we generated phyAp::phyA-nanoLUC (phyA-nLUC) constructs and stably expressed them in the *phyA-211* mutant background, successfully completing the phenotype (*SI Appendix,* Fig. S1). Our data show phyA-nLUC exhibits the expected daily rhythmicity in 10L:14D photoperiods, with phyA-nLUC accumulation at night and depletion during the day (Fig. 1*A* and *SI Appendix,* Fig. S2*A*). These diurnal trends were observed across several reporter lines (*SI Appendix,* Fig. S3). This rhythmic behavior is consistent with earlier findings that both *PHYA* transcripts and phyA protein accumulate during the night, while phyA protein declines during the day due to the relative instability of phyA-Pfr (25, 28, 32). Rhythmicity in phyA levels was previously demonstrated in seedlings transferred from light–dark cycles to continuous light (CL) (30). We observed a low amplitude phyA-nLUC rhythm for 1 to 2 d following transfer to CL, but this rhythm was not sustained over time (*SI Appendix,* Figs. S2*B* and S4). The phyA-nLUC signal depleted to 85% of ZT0 levels after 24 h in CL, and fell to a baseline of ~36% by 110 h (*SI Appendix,* Fig. S2*B*). Our data demonstrate that phyA-nLUC reliably reports phyA levels and offers a quantitative insight into its dynamics.

### Delineating the Contextual Framework for phyA Action.

An initial aim of this study was to elucidate both the experimental and environmental parameters required to elicit phyA action in “canopy shade” (i.e., overhead foliage produced either by an overstory or directly overtopping neighbors). To do this, we used the length of seedling hypocotyls as a quantitative, physiological measure of phytochrome activity (33). Preceding studies suggested that phyA action is prominent in deep shade (e.g., PAR ≤ 15 μmol m^−2^ s^−1^ and R:FR ≤ 0.15) during early seedling development (8, 34). Consistent with these reports, daytime low R:FR (R:FR_0.15_) treatments in 10L:14D photoperiods commencing on day 1 (D1), but not D3, effectively suppresses hypocotyl growth via phyA without impacting germination rates (*SI Appendix,* Fig. S5). All subsequent seedling experiments administered light treatments from D1 postgermination.

We previously established that phyA serves as a critical dawn sensor, primarily due to night-time phyA accumulation and photoactivation upon dawn light exposure (32). Further, the phyB shade avoidance response is known to be circadian gated with peak sensitivity at dusk (35). To assess whether the sensitivity of the phyA activated response also varies with the time of day, we analyzed hypocotyl growth under 10L (PAR = 15 μmol m^−2^ s^−1^, R:FR_7.5_):14D photoperiods when R:FR_0.15_ light was supplied in the morning (ZT0-5), afternoon (ZT5-10), or continuously through the 10-h daylight period (ZT0-10; as illustrated by the schema in Fig. 1*B*). We found that application of R:FR_0.15_ to *phyA-211* seedlings between ZT0-10 or ZT5-10 significantly promoted hypocotyl elongation relative to the R:FR_7.5_ control (Fig. 1*C*). Therefore, as anticipated, eliminating the inhibitory effect of phyA resulted in enhanced growth during times that coincide with the evening peak sensitivity for phyB-mediated shade avoidance (35–37). In contrast, all R:FR_0.15_ treatments, suppressed hypocotyl extension in a phyA-dependent manner in WT, with similar effects observed in *phyB-9* (Fig. 1*C*). We also found R:FR_0.15_ supplied during ZT0-10, ZT0-5, or ZT5-10 elevated levels of phyA-nLUC, *PHYA* mRNA (Fig. 1 *D* and *I*), and *PHYAp::LUC* (*SI Appendix,* Fig. S6*A*), which reports *PHYA* promoter activity. Quantitative phyA-nLUC values for control vs R:FR_0.15_ (ZT0-10) are shown for ZT3, ZT6, and ZT9 replicate time points (*SI Appendix,* Fig. S6*B*). Similar trends were obtained from immunoblot assays, showing a depletion of phyA protein at ZT5 in R:FR_7.5,_ which was less severe in R:FR_0.15_ (Fig. 1*E* and *SI Appendix*, Fig. S9*A*). Therefore, unlike the phyB-SAS, which displays a rhythmic response sensitivity that peaks in the evening (35), exposure to prolonged low R:FR periods can increase phyA production and activity at any time of day. Although phyA levels are low and mainly arrhythmic in continuous light (CL) (*SI Appendix*, Figs. S2*B* and S4), we also found that R:FR_0.15_ was effective in elevating phyA-nLUC levels and suppressing hypocotyl growth in these conditions (*SI Appendix*, Figs. S4 and S7). R:FR_0.15_ does, however, elicit significant hypocotyl elongation in CL, which obscures the phyA HIR response observed in photoperiodic (10L:14D) conditions (*SI Appendix*, Fig. S7).

### PhyA Synthesis and Destruction Dynamics in Canopy Shade.

Dynamic changes in phyA abundance are determined by both destruction- and synthesis-rates (25, 28, 30). To better understand the factors controlling phyA levels throughout the day, we investigated the effects of a proteasome inhibitor (MG132) and a protein synthesis inhibitor (cycloheximide; CHX) on phyA-nLUC levels. First, we observed that abundance of phyA-nLUC in a high R:FR (R:FR_7.5_) photoperiod progressively declined toward a minima at ZT9 compared to predawn levels (ZT23; Fig. 1*F*). Application of MG132 effectively stemmed this daytime reduction in phyA-nLUC, deduced from the comparison of MG132 vs DMSO control, as well as MG132 + CHX vs CHX alone (Fig. 1*F* and *SI Appendix*, Fig. S8). Conversely, CHX treatment resulted in a significant decline in phyA-nLUC levels relative to DMSO or MG132, indicating that phyA is synthesized throughout the day. Under R:FR_0.15_, phyA-nLUC depleted at a slower rate, resulting in higher levels at ZT9 compared to R:FR_7.5_ (Fig. 1 *F* and *G* and *SI Appendix,* Figs. S6 and S8). Reducing the R:FR ratio is expected to increase the proportion of the more stable Pr isoform, potentially reducing proteolysis, while simultaneously enhancing protein (Pr) synthesis (Fig. 1*D*; 17, 19, 28). Nonetheless, the impact of MG132 was slightly more pronounced in R:FR_0.15_ compared to R:FR_7.5_, indicating that proteolysis increases marginally in R:FR_0.15_ (Fig. 1 *F* and *G* and *SI Appendix,* Fig. S8). Prior research has shown that phyA, even in its Pr form, is more quickly degraded in the nucleus than in the cytosol (25). As more nuclear phyA is expected under R:FR_0.15_, this may account for our findings. On the other hand, we observed higher *PHYA* expression in R:FR_0.15_ (Fig. 1*I*), and CHX application was more effective in reducing phyA-nLUC levels in R:FR_0.15_ compared to R:FR_7.5_ (Fig. 1 *F* and *G* and *SI Appendix,* Fig. S8). Thus, R:FR_0.15_ appears to elevate phyA levels in 10 h photoperiods primarily through increased synthesis.

### PhyB Regulation of phyA Dynamics.

As there is evidence for phyA-phyB cross-talk (38–40), we next sought to assess the impacts of the *phyB-9* mutation on phyA. We found that R:FR_0.15_ application to WT was more effective than the *phyB-9* mutation (in R:FR_7.5_) in elevating phyA protein and phyA-nLUC levels (Fig. 1 *G* and *H*). Similar trends were observed for *PHYA* expression, while CHX was also more effective in R:FR_0.15_ (Fig. 1 *G* and *H*). In *phyB-9* seedlings, we observed that CHX unexpectedly caused slight increases in phyA-nLUC after dawn (Fig. 1*H*). Similar outcomes with CHX have been described in past studies and are attributed to its indirect effects, such as suppressing the synthesis of a negative regulator or elevating gene expression (41–43).

The *phyB-9* mutant exhibited a strong response to R:FR_0.15_, in terms of phyA protein and phyA-nLUC levels (Fig. 1*E* and *SI Appendix*, Fig. S9 *A*–*C*). Likewise, R:FR_0.15_ strongly elevated *PHYA* expression in *phyB-9*, while CHX effectively lowered the phyA-nLUC levels in *phyB-9* under the same conditions (Fig. 1*I* and *SI Appendix*, Fig. S9 *A* and *B**)*. Given that phyA typically suppresses its own expression, our data suggest that light-stable phytochromes, other than phyB, likely play a role in regulating *PHYA* synthesis under shaded conditions (44). Additionally, R:FR_0.15_ was more effective in raising phyA protein and phyA-nLUC in *phyB-9* compared to WT (Fig. 1 *E* and *J* and *SI Appendix*, Fig. S9 *A* and *C*). Since CHX mitigated this effect, this again indicates that the increase induced by R:FR_0.15_ is largely attributable to synthesis (Fig. 1*J* and *SI Appendix,* Fig*. S9 D*). Moreover, the data suggest that under shaded conditions, phyB acts to counter the effect of other light-stable phytochromes that actively enhance phyA abundance.

We also found that phyA-nLUC accumulates at a slightly faster rate at night in the *phyB-9* background (Fig. 1*K* and *SI Appendix*, Fig. S10), although this effect might lessen over time. Further, lack of phyB significantly accelerates the decline of the phyA pool immediately following the dark–light transition, as shown in our phyA protein and phyA-nLUC data (Fig. 1 *E*, *H,* and *K*). Given that MG132 application blocks this decline (Fig. 1*H* and *SI Appendix*, Fig. S9*B*), the *phyB-9* mutation appears to increase proteasomal destruction of phyA. In summary, phyB appears to be a potent regulator of phyA, curbing low R:FR-induced *PHYA* expression while enhancing phyA stability and daytime abundance in unshaded, high R:FR, photoperiods.

### PhyA Is a Reliable Sensor of Low R:FR Ratio Canopy Shade.

**Fig. 2.**
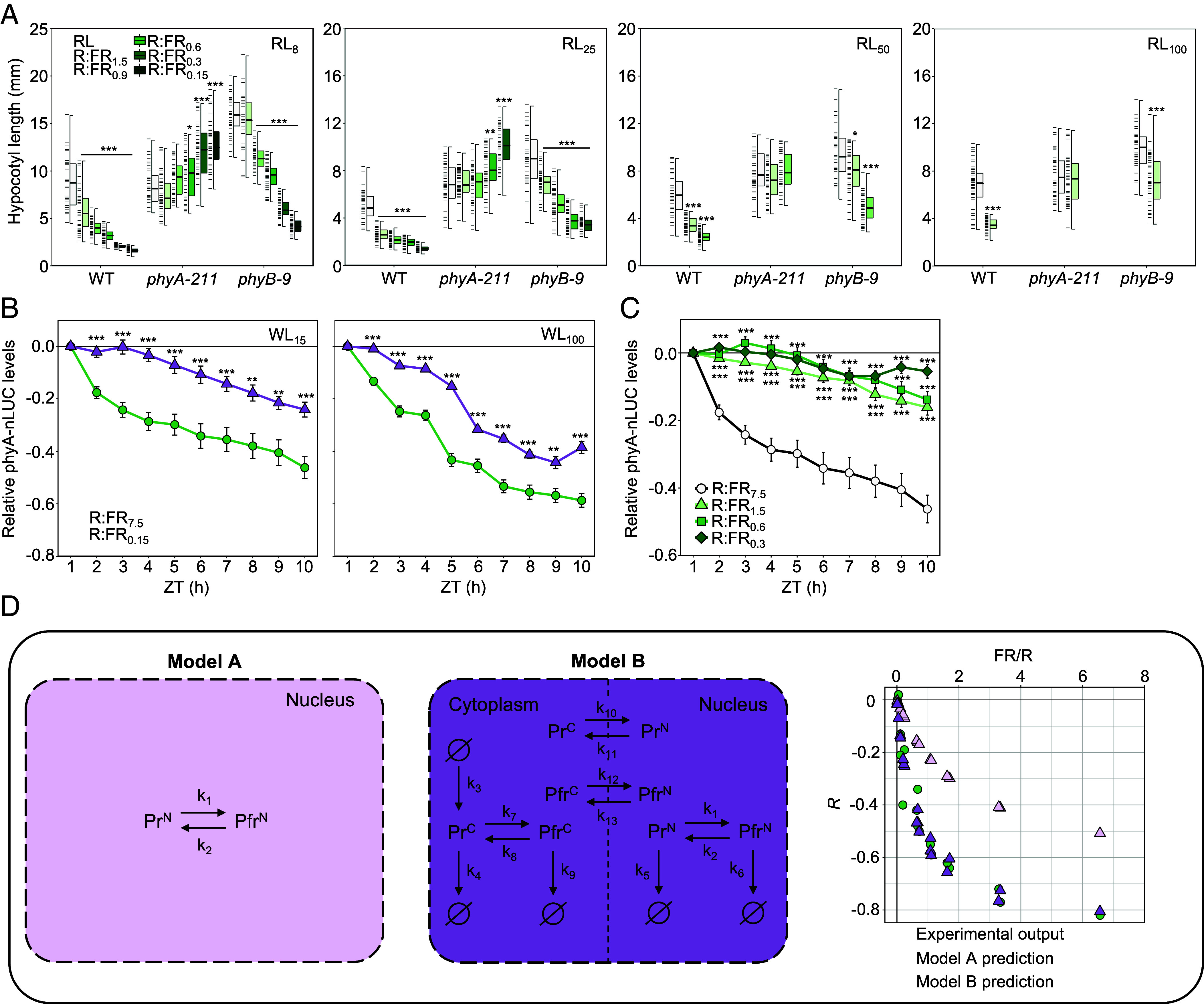
PhyA can respond to subtle changes in the R:FR ratio as predicted by modeling work. (*A*) Hypocotyl lengths of WT, *phyA-211,* and *phyB-9* grown for 6 d in monochromatic red light (RL; 10L:14D) with or without supplementation of FR. Different RL intensities used (8, 25, 50, or 100 μmol m^−2^ s^−1^) are indicated in the *Top Right* corner of each panel. Supplemental FR was applied to each RL condition to reduce the R:FR to 1.5, 0.9, 0.6, 0.3, or 0.15. FR capabilities were limited at higher RL irradiances. Box plots show: median (*Center* line); *Upper* and *Lower* quartiles (box limits); 1.5 × interquartile range (whiskers); individual data points (dashes on the *Left* of boxes, *n* ≥ 25). (*B*) Hourly phyA-nLUC abundance, calculated relative to the first time point after dawn (ZT1) in high (R:FR_7.5_) or low (R:FR_0.15_) R:FR conditions, applied at two WL intensities (PAR = 15 or 100 μmol m^−2^ s^−1^) and (*C*) under different R:FR ratios (R:FR = 7.5, 1.5, 0.6, or 0.3; PAR = 15 μmol m^−2^ s^−1^). Relative change [(value at ZTX − value at ZT1)/value at ZT1] calculated for each seedling, then averaged. Traces show mean signal produced by *n* ≥ 10 plants, error bars show mean ± SEM. Asterisks indicate significant differences from RL controls (*P < 0.05; **P < 0.01; ***P < 0.001), calculated using a Wilcoxon signed-rank test *(A*), and calculated using a Student’s *t* test compared to R:FR_7.5_ controls (*B* and *C*). Each experiment was repeated three times. (*D*) Two alternative models (*A*, *Left*-hand; *B*, *Right*-hand) to explain phyA action. Pr^C^ and Pfr^C^ represent cytoplasmic phyA molecules in their respective states; Pr^N^ and Pfr^N^ show nuclear phyA molecules. Ø represents synthesis and degradation sources, and k_1−13_ reaction parameters. The plot to the *Right* of the models shows the predicted (triangles) and actual (green circles, obtained from experimental work) relative hypocotyl length values [*R* = (value in red + far-red light – average value in red light)/average value in red light], plotted against a function of far-red to red light (FR/R).

Given the complexity of the phyA signaling module and its fluence rate-dependency (7, 17), we next wanted to determine whether phyA can function as a dependable R:FR sensor in scenarios, such as canopy shade, where the R:FR detection capabilities of phyB breakdown (22). In order to be considered a reliable R:FR sensor, we reasoned that phyA must be able to consistently respond to R:FR changes under a variety of light irradiances. We therefore quantified the phyA response across a range of R:FR ratios and at different R light (RL) fluence rates (8, 25, 50, or 100 μmol m^−2^ s^−1^). At RL_8_, we were able to deliver R:FR ratios of 1.5, 0.9, 0.6, 0.3, and 0.15, although limitations in FR capacity of growth chambers meant that the range of R:FR ratio treatments at higher RL fluence rates was more restricted (Fig. 2*A*). Predicted Pfr/Ptot ratios for R:FR treatments are approximately: 0.86 (RL), 0.71 (R:FR_1.5_), 0.63 (R:FR_0.9_), 0.55 (R:FR_0.6_), 0.42 (R:FR_0.3_), and 0.26 (R:FR_0.15_) (*SI Appendix*, Fig. S11*A* and Table S1). For data interpretation purposes, it is important to note that the capability of phyA to operate in a VLFR varies across Arabidopsis accessions and are not detectable in *Columbia*, the accession used in this study (37, 40).

We found that incremental reductions in R:FR gave rise to correlative increases in hypocotyl growth-repression in WT and *phyB-9*, but not in *phyA-211* (Fig. 2*A*). This trend was observed across fluence rates, as was the promotion of phyA-nLUC by R:FR_0.15_ (Fig. 2 *A* and *B*). In contrast to phyA, which responds to a wide range of R:FR ratios, the phyB-driven elongation responses (evident in *phyA-211*) required more severe shade for induction (Fig. 2*A*). Despite being described as a deep-shade detector (6, 8), we observed that a significant phyA-dependent growth inhibition occurred even at mild R:FR ratios, such as R:FR_1.5_ (Pfr/Ptot ~ 0.71; Fig. 2*A* and *SI Appendix*, Fig. S11 *A* and *B* and Table S1). In line with this, application of R:FR_1.5_ significantly increased phyA-nLUC abundance compared to control (R:FR_7.5_) conditions, although lower ratios of R:FR_0.6_ and R:FR_0.3_ did not lead to incremental rises in phyA-nLUC (Fig. 2*C*). This highlights the importance of persistent subtle shade in boosting phyA levels and the phyA response. It also emphasizes that the physiological response is not solely dictated by phyA levels but also involves other elements of the phyA signaling module (17).

**Fig. 3.**
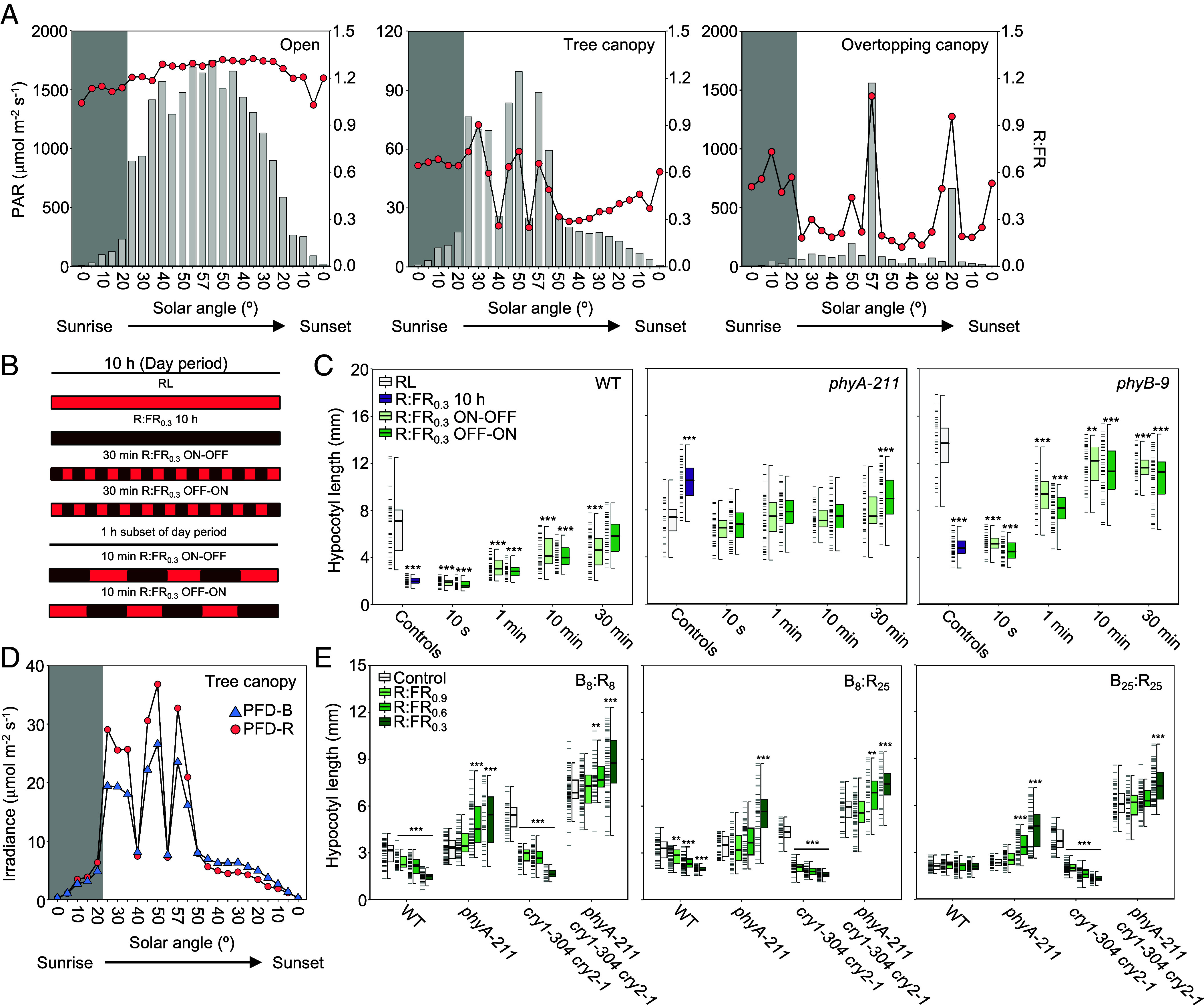
Spectral conditions within canopy shade are suited for phyA operation. (*A*) Comparison of PAR (light gray bars, *Left*-hand y-axis) and R:FR ratio (600 to 700 nm:700 to 780 nm; red circles, *Right*-hand y-axis) measurements across solar angle increments from sunrise to sunset in open (*Left*), tree canopy shade (*Center*), and overtopping canopy (*Right*) environments. (*B*) Visual representation of intermittent low R:FR treatments used in *C*. Each low R:FR period was followed by an equal length period without FR. ON–OFF treatments began the light period with low R:FR and finished without FR, while the opposite was true for OFF-ON. (*C*) Hypocotyl lengths of WT, *phyA-211,* and *phyB-9* seedlings grown in monochromatic RL (25 μmol m^−2^ s^−1^) and either continuously (10 h), or intermittently (10 s, 1 min, 10 min, or 30 min) treated with R:FR_0.3_ throughout the day period. (*D*) Photon flux density (PFD) of blue (B; 400 to 500 nm) and red (R; 600 to 700 nm) irradiances in canopy shade. Spectral data (*A* and *D*) were recorded on 4th June 2022 near Leadburn, Scotland (UK; 55° 46’ 30.0” N, 3° 13’ 13.3” W). Dark gray background on each panel indicates “cloudy” conditions. (*E*) Hypocotyl responses of WT, *phyA-211* single, *cry1-304 cry2-1* double, and *phyA-211 cry1-304 cry1-2* triple mutants to a canopy shade relevant range of R:FR (0.3, 0.6, and 0.9) at combinations of 25 μmol m^−2^ s^−1^ B and R (B_25_:R_25_), reduced (8 μmol m^−2^ s^−1^) B irradiances (B_8_:R_25_) or reduced B and R (B_8_:R_8_). Box plots show: median (*Center* line); *Upper* and *Lower* quartiles (box limits); 1.5 × interquartile range (whiskers); individual data points (dashes on the left of boxes, *n* ≥ 25). Asterisks indicate significant differences from control conditions (*P < 0.05; **P < 0.01; ***P < 0.001). Significance was calculated using a Wilcoxon signed-rank test. Hypocotyl experiments were repeated three times.

Earlier studies have successfully applied modeling approaches as a tool to decipher the complex signaling properties of phyA (17, 19, 21). We aimed to determine if our experimental results align with the established theoretical frameworks describing phyA action. Specifically, we were interested in examining the relationship between irradiance and R:FR ratio as determinants of phyA function. To this end, we calculated the relative hypocotyl length of seedlings grown under descending R:FR (with respect to their lengths in the monochromatic RL), generated through variance in either FR or total (i.e., R + FR) intensity (raw data shown in Fig. 3*A*). We established that there was a very weak correlation between FR (*R*^2^ 0.10, *P* = 0.17) and R (*R*^2^ 0.04, *P* = 0.26) fluence rate with hypocotyl growth inhibition, whereas a strong correlation exists with the R:FR (*R*^2^ 0.91, *P* < 0.001) (*SI Appendix*, Fig. S11*B*).

To enable a direct comparison between model predictions and empirical hypocotyl data (*R*), we created an objective function, under the simple assumption that hypocotyl length linearly increases with phyA-Pfr nuclear levels. Our approach was to develop a series of model variants to test the functionality of key components of the phyA signaling module. The simplest model (Model A; Fig. 2*D*), describes the switching of nuclear phyA between its Pr and Pfr forms. Model B is a more complex model which incorporates key cellular elements of the phyA signaling module. Adapted from Rausenberger et al. (17), Model B includes synthesis and degradation reactions and transport between the nuclear and cytosolic compartments facilitated by the FHY1/FHL transporters (Fig. 2*D*). When compared to the experimental data, Model A predictions follow a similar trend, but fail to match the amplitude of hypocotyl length response (Fig. 2*D*). In contrast, Model B provides an excellent fit to the data (Fig. 2*D*). This indicates that elements of the phyA module in Model B, fulfilled by the photocycle-coupled nuclear shuttling, synthesis, and degradation terms, are required to reproduce the full sensitivity range of hypocotyl growth inhibition observed in the experimental data (Fig. 2 *A* and *D*). Last, we sought to establish the most parsimonious model that could accurately match the data. We found that a simplified model (Model C; *SI Appendix*, Fig. S11*C*), which consolidated parameters yet preserved those related to nuclear flux and degradation rates, also provides a good qualitative match to the data. Our model-based analysis illustrates that steady-state nuclear phyA, which arises from the HIR response mode, provides an effective R:FR ratio sensing mechanism. Support for this concept comes from the constitutively nuclear *pPHYA::PHYA-sGFP-NLS* (phyA-NLS) line, which bypasses the requirement for nuclear shuttling. This line exhibits a constitutively short hypocotyl and is less responsive to reductions in R:FR ratio (*SI Appendix,* Fig. S11*D*). Since phyA-NLS remains capable of detecting changes in R:FR, this suggests nuclear shuttling may serve to enhance the dynamic range of this response.

### Framing phyA Activity in the Context of Natural Shade.

In order to contextualize these findings around the conditions experienced by plants in nature, we analyzed how the R:FR ratio and PAR vary across a day in unshaded “open” conditions and beneath two types of canopy shade: one produced by naturally occurring tree cover and one by “overtopping canopy” closer to ground-level (simulated using four ~30 cm tall *Solanum lycopersicum* plants; images displayed in *SI Appendix*, Fig. S12). PAR ranged from approximately 7 to 1749 μmol m^−2^ s^−1^ in “open,” 1 to 99 μmol m^−2^ s^−1^ beneath the “tree canopy” and 2 to 1561 μmol m^−2^ s^−1^ under the “overtopping canopy” (Fig. 3*A* and *SI Appendix*, Table S2). While PAR diminished with solar angle in all habitats, we found that R:FR gradually increased in canopy shade toward dusk under clear skies. This appears to be a consequence of diminished light-reflectance as irradiances decrease, as there is a more apparent reduction in FR vs R intensity at low solar angles (*SI Appendix*, Fig. S13*A* and Table S2). R:FR ratio ranged between 1.03 to 1.32 (mean = 1.23) in the “open” environment, 0.25 to 0.90 (mean = 0.51) in “tree canopy” and 0.14 to 1.09 (mean = 0.37) in the “overtopping canopy” (Fig. 3*A* and *SI Appendix*, Table S2). The estimated effects of the recorded spectra on the phytochrome-photoequilibria, both in terms of the k_1_/k_2_ transition and Pfr/Ptot ratios, are reported in *SI Appendix*, Fig. S13 *B* and *C* and Table S3. The observed fluctuations in R:FR in both tree and overtopping canopy shade reflect the natural heterogeneity of these mild canopy environments, which causes fluctuations in Pfr/Ptot throughout the day (*SI Appendix*, Fig. S13*C*). This is in part due to cloud cover variation: R:FR was lower beneath canopy shade under clear skies compared to cloudy conditions (gray background in Fig. 3*A* and *SI Appendix*, Table S2) where FR is preferentially absorbed by atmospheric water vapor (45). In addition, we found spikes in PAR and R:FR ratio to regularly occur, most notably at midday (57º) in “overtopping canopy,” which are likely caused by sunlight penetrating through breaks in overstory foliage (sunflecks) (46, 47).

Given that phyA is able to detect mild canopy shade (Fig. 2*A*) but is also reported to rely on continuous FR irradiation to operate in a HIR (14), we decided to test the robustness of phyA signaling during shade interruptions. To do this, R:FR_0.3_, simulating mild shade, was either provided continuously throughout a 10 h day period, or intermittently at 10 s, 1, 10, or 30 min intervals. Two alternating regimes were implemented: one terminating with high R:FR (ON–OFF) and one with low R:FR (OFF–ON) (Fig. 3*B*). We found 10 s pulses were as effective as continuous R:FR_0.3_ in promoting phyA-repression of hypocotyl growth (Fig. 3*C*). 1 min R:FR_0.3_ pulses also elicited a strong phyA response, while longer intervals between treatments were less effective (Fig. 3*C*). Our data indicate that phyA is an adept sensor of R:FR in rapidly fluctuating light conditions, typical of natural mild canopy environments. Interestingly, phyB-driven hypocotyl elongation was only evident in *phyA-211* when 30 min pulses terminated with R:FR_0.3_ or under constant R:FR_0.3_ (Fig. 3*C*). This suggests that, unlike phyA, phyB is a poor detector of intermittent mild shade.

In open environments, and in canopy shade sunflecks (R:FR_0.6−1.1_), the irradiance of R was generally greater than that of blue (B) wavelengths (Fig. 3*D* and *SI Appendix*, Fig. S13*D*). In modest, uninterrupted shade (i.e., R:FR_0.3_) this difference diminished, with R and B fluence rates dropping to around 8 μmol m^−2^ s^−1^ (Fig. 3*D* and *SI Appendix*, Fig. S13*D* and Table S2). Studies have shown that the depletion of B light operating through the cryptochrome photoreceptors, cry1 and cry2, can potentiate the effects of the phyB SAS (48, 49). We therefore wanted to test the impact of low B on the phyA R:FR ratio response in B and R at 8 μmol m^−2^ s^−1^, (B_8_:R_8_), indicative of mild shade, in B_8_:R_25_ (25 μmol m^−2^ s^−1^), and in B_25_:R_25_. We found that addition of B suppressed hypocotyl growth with higher levels (B_25_:R_25_) having the strongest impact, leading to constitutively short hypocotyls across R:FR ratios (Fig. 3*E*). In contrast the *cry1-304 cry2-1* double mutant hypocotyls were longer than WT in all B:R controls, and maintained a strong response to reductions in R:FR ratio that was absent in the *phyA-211 cry1-304 cry2-1* triple mutant (Fig. 3*E*). Thus, in mild shade conditions, cry1 and cry2 appear to operate in concert with phyA to suppress hypocotyl elongation.

### In Mild Canopy Shade phyA Controls Resource Management and Flowering Time.

**Fig. 4.**
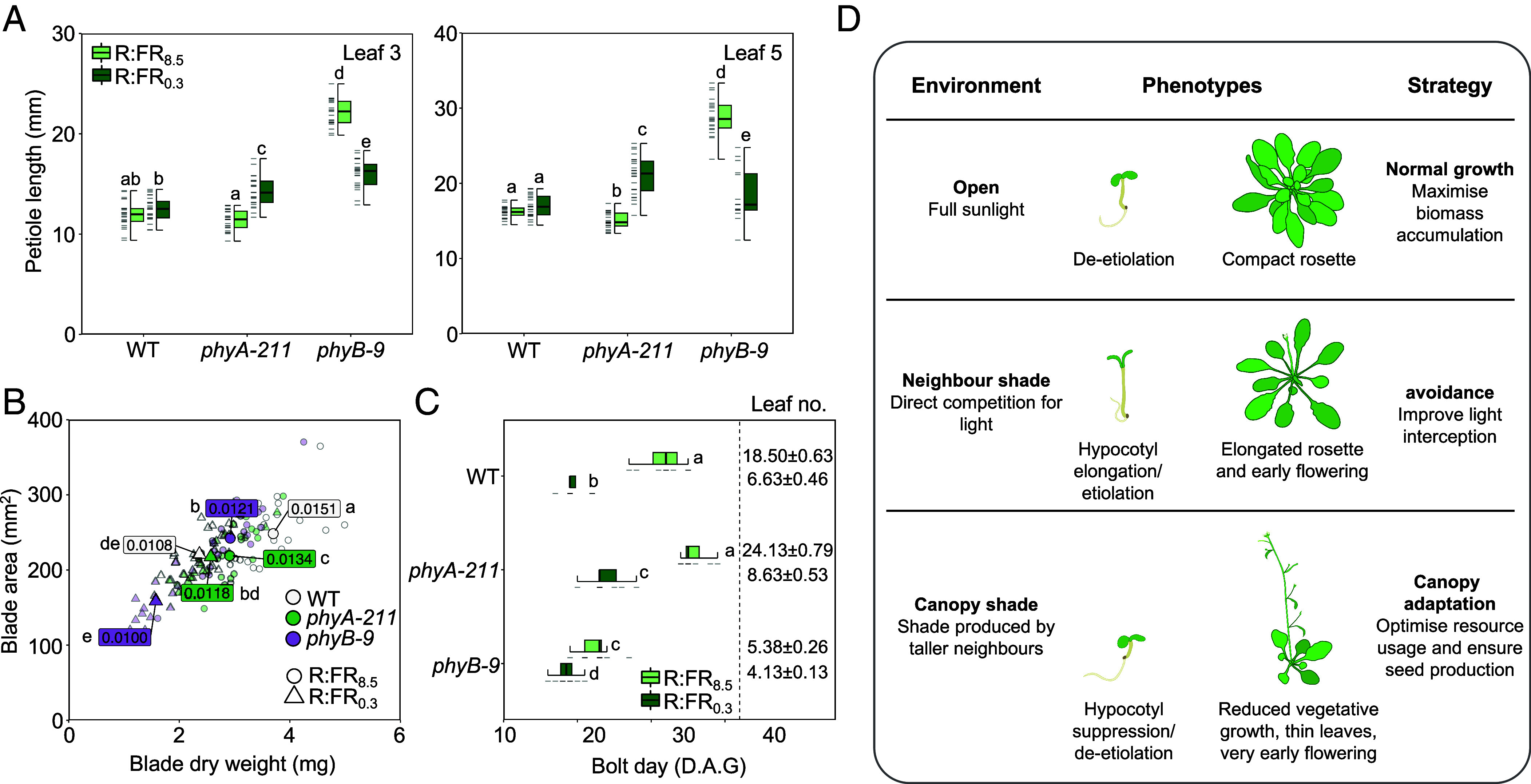
PhyA contributes to a distinct developmental strategy in adult plants that anticipates worsening shade. (*A*) Petiole length of leaf 3 and 5 from WT, *phyA-211,* and *phyB-9* plants at harvest (35-days-old) following growth in WL (100 μmol m^−2^ s^−1^, 12L:12D) without (R:FR_8.5_; Pfr/Ptot ~ 0.80) or with (R:FR_0.3_; Pfr/Ptot ~0.35) FR supplementation. (*B*) Leaf area plotted against dry weight of leaf 5 at harvest. All data points are shown (transparent points), as well as the median for each genotype-treatment combination (solid points). Labels indicate median leaf mass/area (LMA). Significance letters show difference according to LMA; statistical analysis of blade area and dry weight data are included in *SI Appendix*, Table S4. Each treatment was repeated 3 times (*n* = 8), with the combined data from all repetitions being plotted (following outlier exclusion, *n* ≥ 20 for WT and *phyA-211*, but *n* ≥ 14 for *phyB-9* due to reduced leaf production). (*C*) Median bolt day (box and whisker plots) and mean rosette leaf number of plants at bolting (±SEM; *n* = 8). Plants were grown in the same conditions as (*A*) and (*B*) but were not harvested after 35-days in (*C*). x-axis displays day after germination (D.A.G) at which bolting stem first appeared. Box plots show as follows: median (*Center* line); *Upper* and *Lower* quartiles (box limits); 1.5 × interquartile range (whiskers); individual data points (dashes on the *Left* of boxes). Letters (*A–C*) denote statistically indistinguishable groups according to a Kruskal–Wallis test followed by a post hoc Dunn’s Test (Bonferroni correction). (*D*) Summary of the growth strategies adopted by *Arabidopsis thaliana* in different shade and nonshade environments.

Having established that phyA is a sensitive detector of modest canopy shade in seedlings, we subsequently extended our analysis to adult plants. We found exposure to R:FR_0.3_ significantly lengthened petioles of leaves 3 and 5 in *phyA-211* but not WT (Fig. 4*A*). The petioles of *phyB-9*, on the other hand, were markedly shorter in R:FR_0.3_. These data illustrate that under persistent shade phyA effectively restricts the elongation of petioles typically associated with shade avoidance. In R:FR_8.5_, we found all genotypes had similar leaf blade area, while *phyA-211* and *phyB-9* had lower dry weight (Fig. 4*B* and *SI Appendix*, Table S4). Our results indicate both phytochromes contribute to maintaining biomass in unshaded conditions. Lowering the ratio to R:FR_0.3_ led to reductions in WT leaf area and dry weight, with *phyB-9* showing even more pronounced decreases. Thus, in shade the deactivation of phyB and other phytochromes redirects growth and carbon allocation to petioles at the expense of leaves. In comparison to WT and *phyB-9*, the leaves of *phyA-211* are much less sensitive to shade. This contrasts with petiole response, where *phyA-211* enhances shade avoidance type elongation (Fig. 4 *A* and *B*). These findings indicate that phyA does not counteract shade avoidance effects in leaves, but rather has a broader role in maintaining leaf biomass.

Accelerated flowering is a known important feature of phyB SAS, while both phyA and phyB operate in the regulation of photoperiodic flowering (12, 31, 50, 51). Consistent with this, R:FR_0.3_ accelerated flowering in WT, while the constitutively early flowering *phyB-9* showed a more subtle promotion by R:FR_0.3_ (Fig. 4*C*). Conversely, the leaf number at bolting in R:FR_0.3_ was significantly higher in *phyA-211* compared to WT (Fig. 4*C* and *SI Appendix*, Table S5). These data indicate that under mild shade conditions, flowering is hastened by both the deactivation of phyB and the action of phyA. This finding contrasts with the response of hypocotyl growth, where phyA acts to counteract the phyB SAS.

Our data collectively suggest that phyA has a broad and distinct role in plant adjustment to canopy shade, a phenomenon we have named the “Canopy Adaptation Strategy” (CAS). This strategy helps maintain seedling de-etiolation, conserve resources, and accelerate the plant life cycle in environments where the adaptive value of the SAS is diminished (3, 5, 9), thereby improving survival chances (Fig. 4*D*).

## Discussion

PhyA is a key photoreceptor regulating adaptive responses to natural shade. This research has shown that phyA is a sensitive detector of mild canopy shade and is able to reliably detect R:FR ratio even in heterogeneous conditions. PhyA appears to serve an important function in eliciting the canopy adaptation strategy (CAS), which optimizes development in canopy shade conditions by preventing detrimental elongation growth habits in environments when shade cannot be outgrown.

Our study illustrates the highly dynamical properties of phyA which are strongly influenced by light and canopy shade conditions. It is well established that phyA is light labile, and depletes following light induced proteolysis (17, 19, 28). PhyA-nLUC analysis illustrates that proteasomal degradation is, to some extent, counterbalanced *PHYA* synthesis during a 10 h photoperiod (Fig. 1 *A* and *F*). This provides an explanation for the long-standing observation that basal levels of phyA are maintained through the light period (25; *SI Appendix*, Fig. S2). The persistence of phyA through the day may underlie responses such as photoperiodic flowering, particularly in plants grown in unshaded conditions (50, 51).

A key characteristic that emerged from our analysis is that *PHYA* synthesis is incredibly responsive to persistent low R:FR. For instance, unlike the phyB low R:FR response, which is circadian gated (35), phyA levels and activity can be induced by prolonged exposure to low R:FR ratio at any time of day (Fig. 1 *C*, *E,* and *G*). Additionally, our findings demonstrate that even slight decreases in the R:FR ratio, which are too subtle to drive the phyB SAS (Fig. 2*A*; 4, 27), can increase phyA levels and activity. Therefore, beyond its established role as a deep shade sensor, the phyA sensory module appears to be tuned to detect mild canopy shade. We suggest this capability might enable plants to adapt their growth to shade conditions that may intensify over the course of a growing season. The capacity to anticipate increasing shade confers an adaptive advantage to plants, allowing them to complete their lifecycle and set seed before light availability becomes too limiting for growth.

Our data also uncovered modes of crosstalk between phyB and phyA. We found that the loss of phyB greatly enhances the activation of *PHYA* expression and phyA protein accumulation in response to shade light (Fig. 1 *E* and *H* and *SI Appendix*, Fig. S9 *A* and *B*). This observation aligns with previous studies and clarifies why phyA-induced hypocotyl inhibition is more pronounced in *phyB* mutants compared to the WT (Fig. 1*C*; 8, 52). Since phyA typically suppresses its own expression in FR, the increased levels of *PHYA* in *phyB-9* likely results from the action of other light-stable phytochromes (52). Thus, in shade conditions, phyB appears to play an important role in moderating phyA growth suppression by preventing excessive *PHYA* accumulation. Similar to shade, during the night we observe accelerated accumulation of phyA-nLUC in *phyB-9* (Fig. 1*J* and *SI Appendix*, Fig. S10). This may arise, at least in part, from increased activity of PHYTOCHROME INTERACTING FACTOR 4 (PIF4) and PIF5 which promote nocturnal *PHYA* expression through direct binding to the *PHYA* promoter (32). PhyB may moderate this response either by directly inhibiting PIF4 and PIF5 or by promoting the accumulation of EARLY FLOWERING 3 (ELF3), a repressor *PIF4/5* expression and PIF4 action (53, 54).

Interestingly, *phyB-9* also alters the diel regulation of phyA protein abundance. Most notably, we observed enhanced depletion of phyA-nLUC during the daytime in *phyB-9* (Fig. 1*J*), which can be inhibited by the proteasome inhibitor MG132 (Fig. 1*G*). This suggests a role for phyB in decreasing the light lability of phyA potentially through modulation of the proteasomal degradation pathways. Additional research is needed to confirm whether this mechanism is direct or involves posttranscriptional modifications, such as phosphorylation, which is known to modify phyA activity and its susceptibility to proteolysis (55). We hypothesize that connectivity between phyB and phyA likely plays a vital ecological role in varying light environments. For instance, the ability of phyB to exert a moderating effect on *PHYA* expression may help dampen phyA responses in canopy shade. Equally, phyB enhances phyA protein stability in unshaded conditions, which may allow phyA to act in concert with phyB to promote photomorphogenic responses such as seedling de-etiolation.

PhyB is known to be an effective sensor of the R:FR ratio under full sunlight, where Pfr/Ptot is largely determined by the light spectrum and phyB is capable of detecting changes in R:FR indicative of future shading by neighbors (22, 27). However, the reliability of phyB to act as a R:FR sensor diminishes in lower irradiance environments, such as those encountered beneath persistent canopy shade, where the role of phyB as a temperature sensor becomes more prominent (22). In contrast, the limited thermal reversion exhibited by phyA in many species, including various Arabidopsis accessions, may help facilitate accurate R:FR sensing across different climatic conditions (28, 29). However, while the phyA HIR is known to be fluence rate dependent, a property that is critical for detecting sustained low R:FR conditions experienced in canopy shade (Fig. 3*A*), this could reduce the precision of phyA in detecting a range of R:FR ratios (8). We found that the extent of phyA-mediated hypocotyl growth inhibition only weakly correlated with irradiance, but strongly correlated with Pfr/Ptot calculated from R:FR ratio (Fig. 2*A* and *SI Appendix*, Fig. S11 *A* and *B* and Table S1). This suggests that the dependency on fluence rate enables the detection of canopy shade without significantly affecting the ability of phyA to sense the R:FR ratio. Modeling highlighted the relative importance of the phyA light reactions to this capability, including contributions from key elements of the HIR in the R:FR response (Fig. 3*D*). Our simulations indicate that the light reactions are sufficient to trigger a response to shifts in the R:FR ratio (Model A, Fig. 3*D*). Linking the photocycle with the wider elements of the HIR is, however, essential to accurately reproduce the complete sensitivity spectrum to the R:FR ratio seen in the data (Model B, Fig. 3*D*; 17).

Interestingly, we observed that low R:FR light boosts daytime phyA-nLUC primarily via increased synthesis rather than stabilization of phyA (Fig. 1 *E*–*G*). Lowering the R:FR ratio is known to increase the proportion of phyA-Pr, the more stable phyA isomer, yet we observed a slight increase in phyA proteolysis (Fig. 1 *F* and *G*; 17, 28). This increase in proteolysis under low R:FR is likely due to an enhancement in the portion of the phyA pool within the nucleus, where it is targeted for degradation (25, 56). A previous modeling study highlighted the significance of phyA degradation in sustaining the phyA HIR response (17). As such, the combination of increased phyA degradation, coupled with an upregulation of *PHYA* synthesis, may serve to enhance the flux and potentially amplify the phyA response under low R:FR ratios. Furthermore, it is noteworthy that while reducing R:FR to 1.5 stimulated a rise in phyA-nLUC, further reductions to 0.6 and 0.3 did not lead to successive rises in levels (Fig. 2*C*). This again emphasizes the importance of the phyA HIR module elements in delivering the R:FR ratio response (Fig. 2 *A* and *D*). We speculate that elevations in phyA may be important in eliciting a response to very mild shade where nuclear phyA would otherwise be low. Our modeling work (Fig. 2*D*) also suggests that the nuclear concentrating HIR mechanism enables phyA to serve as a sensitive detector of R:FR ratio (*SI Appendix*, Fig. S11*B*).

To contextualize the ecological relevance of our findings, we performed spectral analysis of two mild canopy shade environments across solar angles (*SI Appendix*, Table S3). R:FR ratio varied from 0.25 to 0.9 (estimated Pfr/Ptot ~0.44 to 0.64) beneath the tree canopy and 0.12 to 0.19 (Pfr/Ptot ~0.32 to 0.66) in the overtopping canopy environments (Fig. 3*A* and *SI Appendix*, Tables S2 and S3), ranges within which we found phyA growth suppression to occur (Figs. 2*A* and 3*E* and *SI Appendix,* Fig. S11*B*). Our dataset highlighted the inherent heterogeneity of mild shade environments, which are regularly disrupted by sunflecks (46, 47, 57). When replicating such conditions in our lab experiments, we found that the phyA suppression response is maintained well with brief interruptions in mild shade (e.g., <10 min), whereas the phyB SAS is not induced in intermittent mild shade (Fig. 3*C*). Our results are consistent with previous modeling predictions indicating that phyB activity is dampened by quick transitions between shade and sunflecks, limiting its capability to detect R:FR shifts in canopy environments (22). This is believed to result from the cellular response speed, which may not keep pace with the rapidly changing light conditions. In contrast, phyA is better suited for functioning in heterogeneous light conditions, making it an effective detector of canopy shade.

Our study illustrates that phyA possesses a key role in regulating the response to canopy shade throughout the plant life cycle. Earlier research led to the proposal that the limited seed reserves in species like Arabidopsis are insufficient for shade avoidance responses to occur, instead resulting in a preference for de-etiolation (58). In support of this, we found that even under very mild shade phyA inhibits hypocotyl elongation and maintains de-etiolation (Figs. 1*C*, 2*A*, and 3*E*). This highlights that de-etiolation is likely the default state when seedlings germinate in canopy shade. PhyA-driven de-etiolation in response to R:FR reductions are further facilitated by cry1 and cry2 in the low blue light irradiances typically found in canopy shade (Fig. 3 *D* and *E* and *SI Appendix*, Fig. S13*D*). The adaptive value of this phyA-mediated response has been demonstrated previously, with *phyA* mutants having lower survival rates than WT in dense canopy shade (6). In adult plants, simulation of mild canopy shade from day 7 onward promotes phyA-suppression of petiole growth, which completely masks phyB shade avoidance elongation (Fig. 4 *A* and *B*). These measures may be important for resource conservation and to dampen SAS, which does not confer a competitive advantage under canopy shade environments (5, 9, 59). On the other hand, phyA works in concert with the phyB SAS to accelerate flowering in persistent canopy shade (Fig. 4*C*).

Together, these results support previous studies which suggested that shade-induced elongation growth, such as the SAS, is maladaptive in rosette annuals growing beneath canopy shade (9, 60). We propose that phyA elicits a suite of responses in persistent shade, which we have named the Canopy Adaptation Strategy (CAS). This strategy enhances the survivability of seedlings by facilitating de-etiolation, restructuring carbon resource allocation, and shortening the plant lifecycle to boost the probability of seed-set (Fig. 4*D*). In summary, the phyA photosensory system is precisely calibrated to sense canopy shade, activating the CAS to conserve resources and increase reproductive success.

## Methods

### Plant Materials and Growth Conditions.

*Arabidopsis* (*A. thaliana*) *phyA-211* and *phyB-9* single, the *phyA-211 phyB-9* double (61), as well as the *cry1-304 cry2-1* double and *phyA-211 cry1-304 cry2-1* triple mutant (62) were all of the Columbia-0 (Col-0) ecotype and have been described previously. The *PHYAp::LUC* (firefly LUCIFERASE) construct expressed in the Wassilewskija (Ws) genetic background (63). The *pPHYA::PHYA-sGFP-NLS* (phyA-NLS) line is expressed in the *phyA-201* mutant background (ecotype Landsberg *erecta*; L*er*) (64).

Seeds were surface sterilized using liquid-phase surface sterilization in bleach solution [20% (v/v) domestic bleach, 0.01% (v/v) Triton X-100] and stratified at 4 °C in darkness for 72 h prior to sowing onto ½ Murashige and Skoog (MS) media [1.2% (w/v) agar, pH 5.7, no added sugars]. For adult plant assays, seeds were sown directly onto F2 + S Levington Advance Seed and Modular Compost plus sand soil mix (ICL Group, Israel) following stratification. In all experiments, 24 h prior to experiment start seeds received a 4 h white light (WL; 100 µmol m^−2^ s^−1^) pulse to synchronize germination, followed by 20 h darkness. Plants were grown at 22 °C in all experiments. Seedling experiments were conducted using 10 h light:14 h dark (10L:14D) photoperiods, unless otherwise stated. Adult plant assays used a 12L:12D photoperiod and seedlings were grown using standard WL conditions (100 µmol m^−2^ s^−1^, R:FR = 8.5) and under a clear plastic lid for 6 d to ensure even germination. On day 7, the lid was removed, seedlings were thinned, and trays were transferred to the relevant condition.

WL was provided in Percival I30-BLL growth chambers (CFL Plant Climatics, Germany) by Luxline Plus F18W/840 fluorescent tubes (Sylvania, Newhaven, UK; spectrum shown in *SI Appendix*, Fig. S14*A*). Neutral density filters (LEE Filters Worldwide, UK) were used to adjust WL irradiance to desired levels. Supplementary FR (peak ~730 nm) was provided by OLSON 150 6+ Series FR LED strips (Intelligent LED Solutions, UK), filtered through LEE 120 Deep Blue color filter (LEE Filters Worldwide) to remove excess R light. For experiments under monochromatic R ± FR and R + B ± FR, EB2-NE-PB Cooled Incubators (Snijders Labs, The Netherlands) custom fitted with “blue” (peak ~456 nm), “deep red” (peak ~680 nm) and “far-red” (peak ~782 nm) GreenPower LEDs (Philips, the Netherlands) were used (spectral outputs shown *SI Appendix*, Fig. S14*B*). Light conditions were determined using a LI-180 Spectrometer (LI-COR, NE). The total photosynthetic active radiation (PAR), R:FR ratios, and the irradiances (photon flux density; PFD) of blue (B; 500 to 600 nm), green (G; 600 to 700 nm), red (R; 600 to 700 nm), and far-red (FR; 700 to 780 nm) wavebands used in experimental work are listed in *SI Appendix,* Table S6. R:FR was quantified as the ratio between wavelengths from 600 to 700 nm: 700 to 780 nm. While narrower wavelength ranges are (e.g., 640 to 700 nm: 700 to 760 nm and 640 to 670 nm: 720 to 750 nm) are sometimes favored as they lie closer to the absorption maxima of Pr (~660 nm) and Pfr (~730 nm) (12), we found that narrow waveband ranges resulted in lower R:FR calculations under broad-spectrum natural environments. This resulted in skewed Pfr/Ptot estimations when spectral data was integrated with phytochrome photoconversion cross-sections (65) compared to when R_600−700_:FR_700−780_ was used (*SI Appendix*, Tables S3 and S7).

### Morphological Data Collection.

Hypocotyl length data were collected from 6-d-old seedlings: images of seedlings were taken 1 h prior to dawn on their 7th treatment day and hypocotyl lengths were measured using ImageJ (https://imagej.net/ij/). Each treatment was repeated 3 times where *n* ≥ 25 for each genotype. Adult plants were harvested 1 h before dawn on day 36 of treatments (Fig. 4 *A* and *B*). Leaf 3 and 5 were removed, blades were severed from petioles and images were captured for respective measurements. All images were analyzed using ImageJ. Blades of leaf 5 were wrapped in preweighed aluminum foil and dried at 80 °C for 24 h before dry weight measurements were obtained. Leaf Mass per Area (LMA; mg/mm^2^) was calculated as leaf mass/leaf surface area. Time to flower (Fig. 4*C* and *SI Appendix,* Table S5) was taken as the day at which a bolting stem first became visible, at which point rosette leaf number was also recorded (Fig. 4*C*).

### RNA Extraction and cDNA Synthesis.

Seedlings were grown for 6 d in 10L:14D (PAR = 15 µmol m^−2^ s^−1^, R:FR = 7.5; 22 °C). At the start of their 7th day, seedlings were exposed to the relevant light conditions [WL (R:FR = 7.5) or FR (R:FR = 0.15)] where they were maintained until the relevant time points (ZT1, 3, 8, or 10), at which point seedling tissue was collected, snap frozen in liquid N_2_ and stored at −80 °C. Frozen tissue was ground, then 300 µL of preheated (60 °C) RE buffer (0.1 M Tris pH 8.0, 5 mM EDTA pH 8.0, 0.1 M NaCl, 0.5% SDS) + 1% β-mercaptoethanol was added and samples were vortexed until homogenized. 300 µL of a 1:1 acidic phenol:chloroform mix were added to the sample and vortexed further, before being transferred to ice. Samples were subsequently centrifuged at 4 °C for 15 min at maximum speed. Up to 300 µL of the supernatant was transferred to a new collection tube, mixed gently, then stored at 4 °C to induce the precipitation of nucleic acids. After 15 min, samples were centrifuged at 4 °C for 30 min at maximum speed to pellet all nucleic acid precipitate. Supernatants were then discarded and pellets were gently washed with 300 µL of 70% ethanol (w/v), followed by a further centrifugation for 5 min at max speed (4 °C). All traces of ethanol were removed and the pellet was dissolved in 50 µL of nuclease-free water. cDNA synthesis was conducted on extracted RNA using the qScript cDNA synthesis kit (Quantabio, MA) as per the manufacturer’s instructions. cDNA concentration was quantified using an ND-1000 NanoDrop (Thermo Fisher Scientific, MA).

### qRT-PCR.

SYBR Green qPCR master-mix (Thermo Fisher Scientific) was used following the manufacturer’s protocol. Relative transcript abundance of *PHYA*, normalized to *PROTEIN PHOSPHATASE 2A* (*PP2A*), was calculated using the ΔΔCt-method. Gene-specific oligonucleotides for *PHYA* (FW-GTTTGGGACTGAGGAAGATGTG; RV-CTTTTGGGGACTACTTGTTTGC) and reference gene *PP2A* (FW-TAACGTGGCCAAAATGATGC; RV-GTTCTCCACAACCGCTTGGT) were used to quantify transcript levels using the 2^−ΔΔCt^ method. For each data point, three technical and three biological replicates were performed.

### Western Blot Analysis.

Each sample consisted of 0.3 g of *Arabidopsis* seedling tissue grown under 10L:14D (PAR = 15 µmol m^−2^ s^−1^, R:FR = 7.5; 22 °C) for 6 d. Tissue was harvested on day 6 either immediately prior to the dark–light transition at ZT0 on (constituting the darkness time point) or at midday (ZT5) following exposure to R:FR_7.5_ or R:FR_0.15_. 200 μM CHX was added at ZT0, DMSO used as the control. Samples were added to lysis buffer [125 mM Tris-HCl (pH 7.5); 0.25 mM EDTA (pH 7.5), 150 mM NaCl, 5% glycerol, 0.1% Triton X-100, 0.5% NP-40]. Samples were centrifuged at 12,000 rpm for 10 mins at 4 °C and then transferred to lysate mix with 1× SDS loading buffer (containing 0.1 M DTT) at a 1:1 volume ratio. Samples were incubated at 80 °C for 10 mins, followed by a centrifugation step, prior to western blotting. To detect phyA protein, an anti-Phytochrome A antibody (PHYTOAB, PHY1907) was used at 1:1,000. Anti-Actin antibody (Abcam, ab190301) was used at 1:3,000 as internal control. Secondary antibodies (1:3,000, anti-mouse IgG; cell signaling, 7,076 s) were used for the ECL Prime Western Blotting System. Signals were detected using an Azure 300 imager (Azure Biosystems, CA) with ECL prime western blotting detection reagents (Cytiva, MA).

### In Planta Bioluminescence Assays.

Surface-sterilized seeds were individually sown into wells of white LUMITRAC 96-well plates (Greiner Bio-One, Austria) containing 200 µL of solid ½ MS media prior to stratification for 72 h at 4 °C. Seeds received a germination pulse (as described above) before a 7-d entertainment period began using 15 µmol m^−2^ s^−1^ WL (10L:14D). After this, 50 µL of 2% (v/v) Nano-Glo® (Promega, WI) or 100 µL of 50 mM Luciferin (Sigma-Aldrich, MI) was applied per well for nLUC and LUC assays, respectively. Plates were sealed with TopSeal-A plus (PerkinElmer, MA) and transferred to a Tristar^2^ plate reader (Berthold, Germany) for 5 to 13 d, where bioluminescence was measured every hour with an integration time of 1.5 s per well. In between readings, seedlings were maintained in 15 µmol m^−2^ s^−1^ WL (Luxline Plus F18W/840, Sylvania) with (R:FR_0.15_) or without (R:FR_7.5_) supplemental FR (OLSON 150 6+ Series LED; Intelligent LED Solutions).

### Cycloheximide and MG132 Treatment.

Seedlings were initially grown in a 96-well plate for 4 d, following the protocol described for the bioluminescence assay. On the 5th day, seedlings were treated with the Nano-Glo® substrate and subjected to plate reader measurements. At the 9-d-stage, just before the final time point of the night period (ZT23), seedlings were treated with 200 µM CHX (C1988; Sigma-Aldrich) and/or 50 µM MG132 (474787; Sigma-Aldrich), with DMSO used as control. Bioluminescence readings from treated seedlings were subsequently taken every hour during the following light period (ZT1-10), with an integration time of 1.5 s per well.

Methods to be found within the *SI Appendix* are as follows: phytochrome photoequilibrium calculations; spectral data collection; plasmid construction and plant transformation; model development; computational resources and data analysis.

## Supplementary Material

Appendix 01 (PDF)

## Data Availability

All study data are included in the article and/or supporting information.
