# Supplementary material for: Arabidopsis thaliana phytochrome A sensory properties in canopy shade
Source: Proc Natl Acad Sci U S A. 2026 Jan 27;123(5):e2512201123. doi: 10.1073/pnas.2512201123 (PMC12867752; doi:10.1073/pnas.2512201123)
Supplement: Supplementary file 1 — Appendix 01 (PDF) [file pnas.2512201123.sapp.pdf]

PNAS

Supporting Information for
Arabidopsis thaliana Phytochrome A Sensory Properties in Canopy Shade

Philip Prizeman-Green[†, a], Marissa Valdivia-Cabrera[†, a], Mengke Zhou[a], Ramon Grima[a] and Karen J. Halliday[*, a]
[†] Authors have made equal contributions
[a] School of Biological Sciences, University of Edinburgh, Edinburgh 3H9 3BF, United Kingdom
* Corresponding author (Karen J. Halliday)

Email: karen.halliday@ed.ac.uk

This PDF file includes:

Supporting text - SI Methods
Figures S1 to S14
Tables S1 to S8
SI references

Supporting Information Text

SI Methods

Phytochrome photoequilibrium calculations

To calculate Pfr/Ptot, we took the known type I phytochrome k_1 (Pr → Pfr) and k_2 (Pfr → Pr) conversion rates (originally from oat phyA; 1) at each wavelength (nm) and multiplied these by the actual irradiance (μmol m^{-2} s^{-1}) recorded to produce a transition rate (min^{-1}). To calculate the total transition rate for each phytochrome form across the total wavelength range measured, each wavelength interval (e.g. 381 nm - 380 nm) was then multiplied by the average transition rates at the two endpoints of the interval. These values were then summed to get total k_1 and k_2 rates. This prevents issues arising from non-consistent wavelength intervals recorded by a spectrophotometer. The Pfr/Ptot was then estimated using the sum of these transition rates as follows: Pfr/Ptot = $\sum k_1$ / [$\sum k_1 + \sum k_2$]. Calculations were conducted using MATLAB (vR2024a) with scripts kindly provided by Dr Johanna Krahmer.

Spectral data collection

Spectral data from an un-shaded 'open' and woodland 'canopy shade' environment was collected on 4th June 2022 near Leadburn, Scotland (UK; approximately 55° 46' 30.0" N, 3° 13' 13.3" W). This site was away from artificial street lighting and free of confounding obstructions to the path of light from the sun (i.e. from buildings and hills). Measurements were taken at 5º increments in solar angle (relative to the horizon) from sunrise to sunset (both 0º), as well as one recording at the solar zenith (57º) . The corresponding time for each solar angle was determined for the nearest available location (Leadburn, UK) using an online resource (https://www.timeanddate.com/sun/). The presence/absence of cloud cover at each measurement point was noted: 'cloudy' conditions occurred when clouds visibly blocked the path of sunlight; other time points are marked 'clear'. Spectral readings, measured as photon flux density (PFD; recorded in μmol m^{-2} s^{-1}) across 1 nm increments from 380-780 nm, were collected using a LI-180 spectrometer (LI-COR, NE, USA). In addition, PAR as well as PFD-B (400-500 nm), -G (500-600 nm), -R (600-700 nm) and -FR (700-780 nm) were also obtained and used to calculate photon ratio measurements. Each measurement is an average of 6 readings taken across 1 min at 10 s intervals to account for small fluctuations in spectral composition.

Plasmid construction and plant transformation

To generate the phyA-nLUC (phyAp::phyA-nanoLUCx3FLAGx10His) transgenic line, the phyA promoter (2141 bp upstream of the inferred initiation codon) was amplified with Q5 High-Fidelity DNA polymerase (New England Biolabs, MA, USA) from the Arabidopsis Col-0 genome. The coding region was obtained from mRNA extracted using the RNAeasy mini kit (Qiagen, Germany). cDNA was synthesised using the qScript kit (Quantabio, MA, USA). The nLUC tag (NLUCx3FLAGx10His; NL3F10H) was amplified from the pET28::NL3F10H construct (2). All parts were recombined in the pGWB601 vector (3) using the Gibson Assembly method (NEBuilder HiFi DNA Assembly master mix kit). Primers for plasmid construction are listed in *SI Appendix*, Table S8. After sequence verification, *Escherichia coli* (DH5α) cells were transformed via heat-shock, following the Cold Spring Harbor Laboratory Protocol 1.24 (2006), and selected on spectinomycin plates. Plasmids were purified using the Monarch Plasmid Miniprep kit (New England Biolabs). Each cloned intermediate nucleotide sequence was verified through Sanger sequencing (Edinburgh Genomics, UK). *Agrobacterium tumefaciens* (strain ABI1, resistant to kanamycin and chloramphenicol) were transformed using the freeze and thaw method (4). Plant transformation was carried out using the floral-dip method (5). Segregation analysis of lines expressing the phyA-nLUC construct in the *phyA-211* mutant and the *phyA-211 phyB-9* double mutant background was

performed using ½ MS plates containing 10 µg/ml Bialaphos. Phenotypic complementation assays were subsequently performed on homozygous lines, with *phyA-211* correctly expressing phyA-nLUC displaying a wild-type phenotype (*SI Appendix*, Fig. S1) and *phyA-211 phyB-9* for a *phyB-9* mutant.

Model development

Model A. This consists of two reactions and two species (Pr^N and Pfr^N). The rate at which Pr^N changes to Pfr^N is equal to 5.31 Nr + 0.82 Nfr while the rate at which Pfr^N changes to Pr^N is equal to 0.04 Nr + 1.70 Nfr where Nr and Nfr are the intensities of red and far-red light, respectively. The four constants in these expressions were extracted from (1) assuming the dominant wavelength of red light is 666 nm and that of far-red light is 730 nm. The standard deterministic rate equations (6) for this model were solved for the concentration of Pfr^N (denoted as $[Pfr^N]$) as a function of the light intensities. Assuming the hypocotyl length is proportional to $[Pfr^N]$, it then follows that the quantity R = (L – L0)/L0 in Fig. 2 *D* of the main text is computed as ($[Pfr^N]$- $[Pfr^N]^*$)/ $[Pfr^N]^*$ where $[Pfr^N]^*$ is the concentration of Pfr^N when the far-red light intensity Nfr is set to 0. Specifically, R is found to be given by equation 1.

1. $R = -\frac{169.382\, Nfr}{252\, Nfr + 535\, Nr}$

Model B. This consists of 13 reactions and four species (Pr^C, Pfr^C, Pr^N and Pfr^N). The rates of the two reactions that change Pr to Pfr and vice versa (in cytoplasm or nucleus) after red or far-red absorption are as in Model A. We also set the transport rates from nucleus to cytoplasm (and vice versa) to be the same for Pr and Pfr. The decay rates of Pr and Pfr are also the same in the nucleus as in the cytoplasm. These assumptions considerably reduce the size of parameter space and hence simplify the optimization of the model to the data. The best fitting of the model's prediction of R to the experimental data was implemented using the following procedure. We solve the rate equations for the model to obtain the concentration of Pfr^N and hence calculate R = (L – L0)/L0. The rate parameters which provide the closest fit were obtained by minimising the sum of the square of the differences between the experimental and predicted values of R where the sum is taken over the 22 measured tuples of Nfr, Nr and R values.

Model C (*SI Appendix*, Fig S11 *C*). This consists of 6 reactions and two species (Pr^N and Pfr^N). The rates of the two reactions that change one species to another after R or FR absorption are as in Model A. There are now four other rates (two for the production reactions and two for the removal reactions) which are unknown. We solve the rate equations for this model to obtain the concentration of Pfr^N and hence calculate R = (L – L0)/L0. The rate parameters which provide the closest fit were obtained by minimising the sum of the square of the differences between the experimental and predicted values of R where the sum is taken over the 22 measured tuples of Nfr, Nr and R values. Note that for simplicity and ease of finding the optimal parameter values, we reduced the size of parameter space by assuming that the rate of production of Pr^N is the same as that of Pfr^N.

Computational resources and data analysis

nLUC and LUC time-course data was subject to detrending and normalisation following the Amp & Baseline detrending method implemented in the BioDare2 platform (www.biodare2.ed.ac.uk) (7). This process involves removing low-frequency baseline trends using a local linear regression with a Gaussian kernel. Subsequently, detrended data are converted to absolute values to derive amplitude estimates. A second local linear regression is applied to capture the gradual temporal trend. The original baseline-detrended signal is then divided by this smoothed amplitude trend to yield a normalised time series. "To [-1,1]" normalisation rescales the timeseries values to a mean value of 0 and oscillations in the range [-1,1]. This method of removing baseline trends and

normalising amplitude variations, enables accurate comparison of cyclical patterns across different samples.

Primers were designed utilising the 'Primer Wizard' tool available on the Benchling platform (www.benchling.com). Data were plotted using Rstudio (v.2024.04.1+748) and GraphPad Prism (v8.0) for Windows. Statistical methods are included in the figure legends. The model parameters that best fit the data were determined using Mathematica, Version 14.1 (Wolfram Research, Inc., Champaign, IL).

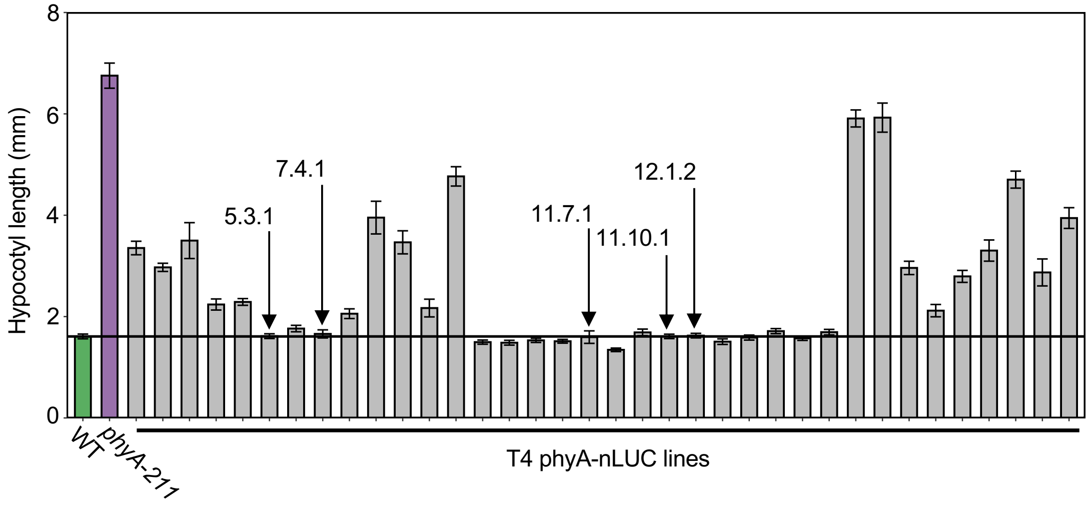

Fig. S1. T4 selection of phyA-nanoLUC lines for phenotypic complementation of the *phyA-211* phenotype. Hypocotyl analysis of 36 phyAp::phyA-nanoLUC (phyA-nLUC) homozygous lines (grey bars) scored for their ability to restore the wild-type (WT; Col-0, green bar) phenotype of the *phyA-211* (purple bar) mutant. The phyA-nLUC construct was expressed in a *phyA-211* background with homozygous lines selected previously for resistance to bialaphos. Arrows indicate the first lines selected for analysis of PHYA protein dynamics. Bars show mean ± SEM ($n \geq 22$). Horizontal black line represents the mean length of WT seedlings. Seedlings were grown for 6 days on ½ MS solid media at 22°C in 15 µmol m^{-2} s^{-1} (10L:14D) of white light supplemented with FR (R:FR = 0.15).

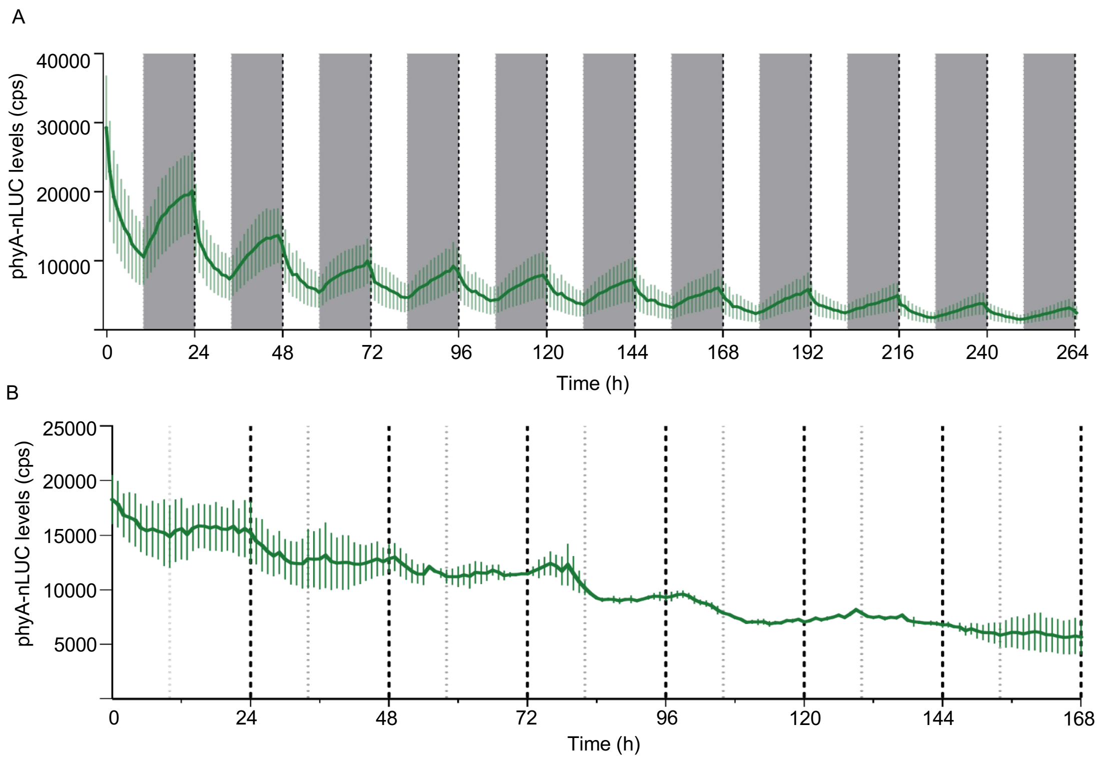

Fig. S2. Bioluminescence analysis of phyA-nLUC under diurnal conditions (*A*) and constant light (*B*). Dynamics of entrained phyA-nLUC seedlings grown in R:FR$_{7.5}$. Background colours of each panel correspond to R:FR$_{7.5}$ (white) and night (grey) periods. Bioluminescence was measured at 1 h intervals. Traces show mean signal produced by *n* ≥ 10 plants; error bars show ± SEM.

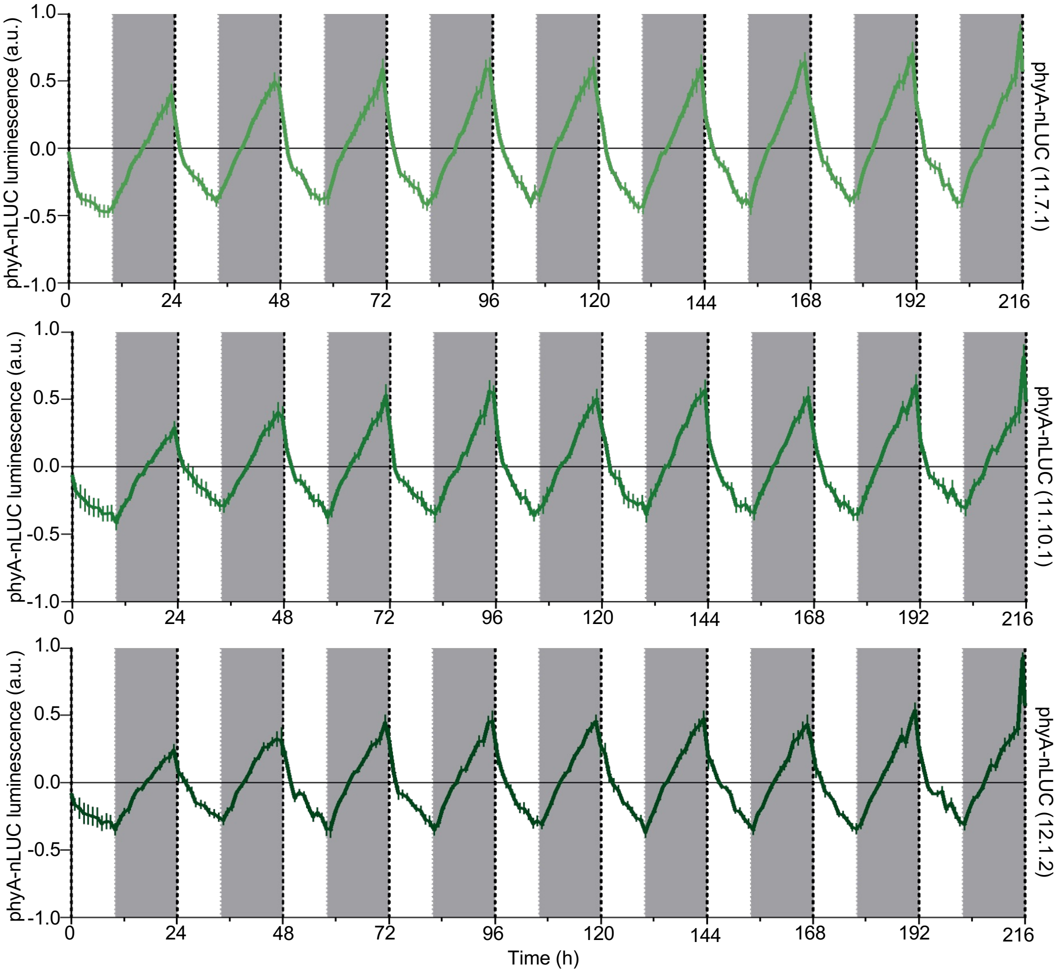

Fig. S3. PhyA-nLUC diurnal dynamics tested on three different homozygous lines. Bioluminescence of phyA-nLUC (arbitrary units, a.u.) of entrained seedlings grown in R:$FR_{7.5}$. Background colours of each panel correspond to R:$FR_{7.5}$ (white) and night (grey) periods. Lines are labelled on the right side of each panel. Bioluminescence was measured at 1 h intervals. Traces show mean signal produced by $n \geq 10$ plants; error bars show ± SEM.

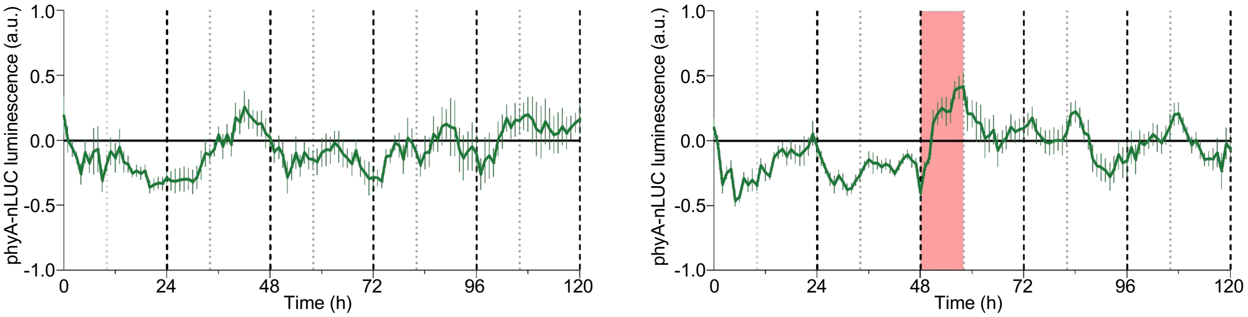

Fig. S4 Low R:FR treatment increases phyA-nLUC levels in constant light. Bioluminescence of phyA-nLUC (arbitrary units, a.u.) from entrained seedlings grown in R:$FR_{7.5}$ and transfer to constant light. Left-hand panel shows rhythms in constant R:$FR_{7.5}$, and right-hand panel shows R:$FR_{7.5}$ plus exposure to low R:FR (0.15) at 48 h. Background colours of each panel correspond to R:$FR_{7.5}$ (white) and R:$FR_{0.15}$ (red) periods. Bioluminescence was measured at 1 h intervals. Traces show mean signal produced by $n \geq 10$ plants; error bars show ± SEM.

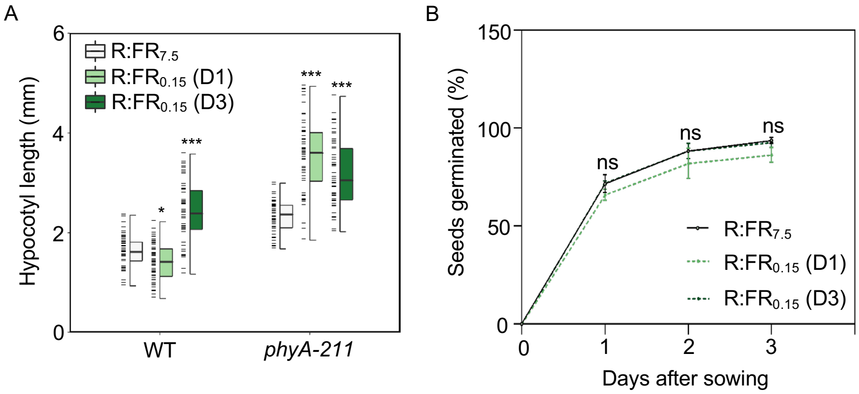

Fig. S5 Hypocotyl and germination responses to application of low R:FR from day 1 or day 3. (*A*) Hypocotyl lengths of 6-day-old WT and *phyA-211* seedlings grown in R:$FR_{7.5}$ (predicted Pfr/Ptot ~0.80) or R:$FR_{0.15}$ (~0.24) commencing either on day 1 (D1) or D3 post-germination pulse. Germination pulses were initiated 24 h prior to treatment commencement and consisted of 4 h 100 µmol m^{-2} s^{-1} WL (R:FR = 7.5) followed by 20 h darkness (22ºC). (*B*) Germination rates of WT and *phyA-211* seedlings in R:$FR_{7.5}$ (solid black line), R:$FR_{0.15}$ provided from day 1 (D1; dotted green line) or D3 (dotted black line) post-germination pulse. Data in both *A* and *B* were produced in the same experiment. PAR = 15 µmol m^{-2} s^{-1}; 10L:14D. (*A*) Box plots display the median (centre line); upper and lower quartiles (box limits); 1.5 x interquartile range (whiskers); individual data points (dashes on the left of boxes, n ≥ 25). Asterisks indicate significant differences from controls (*P < 0.05; **P < 0.01; ***P < 0.001) calculated using a Wilcoxon signed-rank test. (*B*) Points indicate the mean; error bars show the SEM (n ≥ 25). Statistical analysis was made using the Tukey's Method (ordinary one-way ANOVA; P > 0.05, ns).

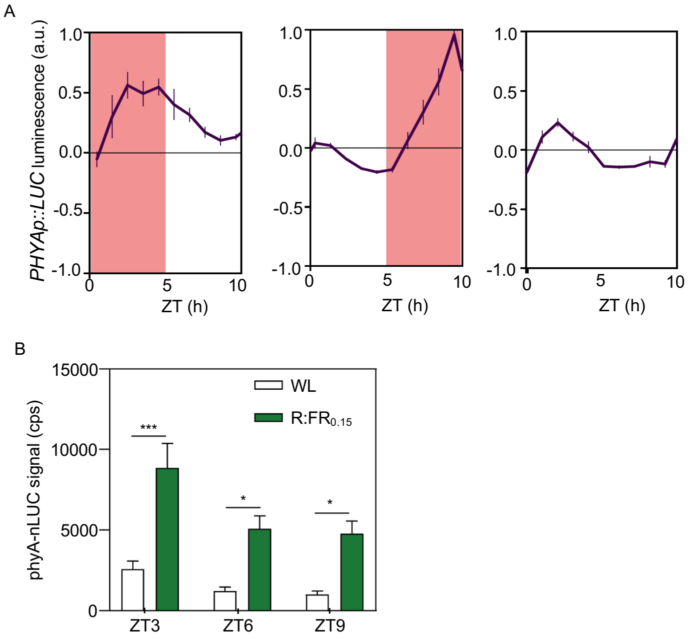

Fig. S6. Low R:FR increases *PHYA* expression and phyA abundance throughout the day. (*A*) Bioluminescence of *PHYAp::LUC* (arbitrary units, a.u.) from seedlings grown in R:FR$_{7.5}$ and exposed to low R:FR$_{0.15}$ in the morning (ZT0-5; left-hand panel), evening (ZT5-10; middle panel) or kept in R:FR$_{7.5}$ (right-hand panel). Background colours of each panel correspond to R:FR$_{7.5}$ (white) and R:FR$_{0.15}$ (red) periods. Bioluminescence was measured at 1 h intervals. Traces show mean signal produced by $n \geq 10$ plants; error bars show ± SEM. (*E*) phyA-nLUC signal strength at ZT3, 6 and 9 in R:FR$_{0.15}$ compared to the same time points in R:FR$_{7.5}$. Bars indicate the mean; error bars show ± SEM ($n \geq 10$). Asterisks indicate significant differences from controls (*$P < 0.05$; **$P < 0.01$; ***$P < 0.001$) calculated using a Student's t-test.

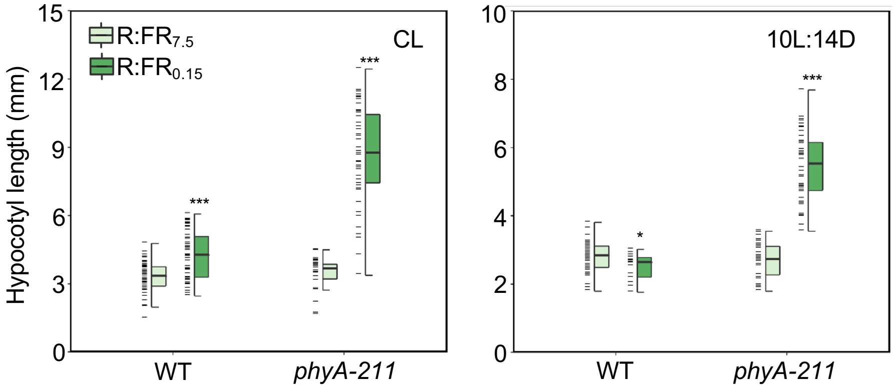

Fig. S7. PhyA-mediated hypocotyl suppression is stronger in photoperiodic conditions. Hypocotyl lengths of WT and *phyA-211* seedlings grown in R:FR$_{7.5}$, or R:FR$_{0.15}$ in constant light (CL; left-hand panel) or during a 10L:14D photoperiod (right-hand panel). PAR = 15 μmol m^{-2} s^{-1}. Each box plot shows as follows: median (centre line); upper and lower quartiles (box limits); 1.5 x interquartile range (whiskers); individual data points (dashes on the left of boxes, $n \geq 25$). Asterisks indicate significant differences from controls (*$P < 0.05$; **$P < 0.01$; ***$P < 0.001$) calculated using a Wilcoxon signed-rank test.

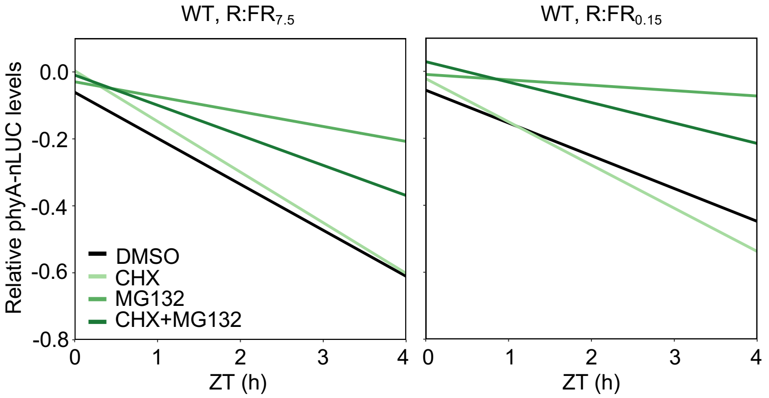

Fig. S8. Influence of inhibitors on rates of phyA-nLUC depletion in high or low R:FR conditions. Rate of change in hourly phyA-nLUC abundance expressed in WT seedlings treated with 50 µM MG132 and/or 200 µM CHX to block either proteasomal degradation, translation or both; DMSO alone was used as a control. Relative phyA-nLUC levels for the first 4 hours following the dark-light transition (ZT0) into a high ($R:FR_{7.5}$, left-hand panel) or low ($R:FR_{0.15}$, right-hand panel) day period, calculated with respect to the point of pre-dawn inhibitor application (ZT23), were used. Relative change for each treatment = (value at ZTX - value at ZT23) / value at ZT23.

Equations and values are as follows:

$R:FR_{7.5}$, DMSO: y = -0.1374x - 0.06081; degradation rate = -0.137
$R:FR_{7.5}$, CHX: y = -0.1513x + 0.003494; degradation rate = -0.151
$R:FR_{7.5}$, MG132: y = -0.04452x - 0.02949; degradation rate = -0.045
$R:FR_{7.5}$, CHX + MG132: y = -0.08990x - 0.009456; degradation rate = -0.089
$R:FR_{0.15}$, DMSO: y = -0.09795x - 0.05622; degradation rate = -0.098
$R:FR_{0.15}$, CHX: y = -0.1290x - 0.02183; degradation rate = -0.129
$R:FR_{0.15}$, MG132: y = -0.01609x - 0.009286; degradation rate = -0.016
$R:FR_{0.15}$, CHX + MG132: y = -0.06104x + 0.02885; degradation rate = -0.061

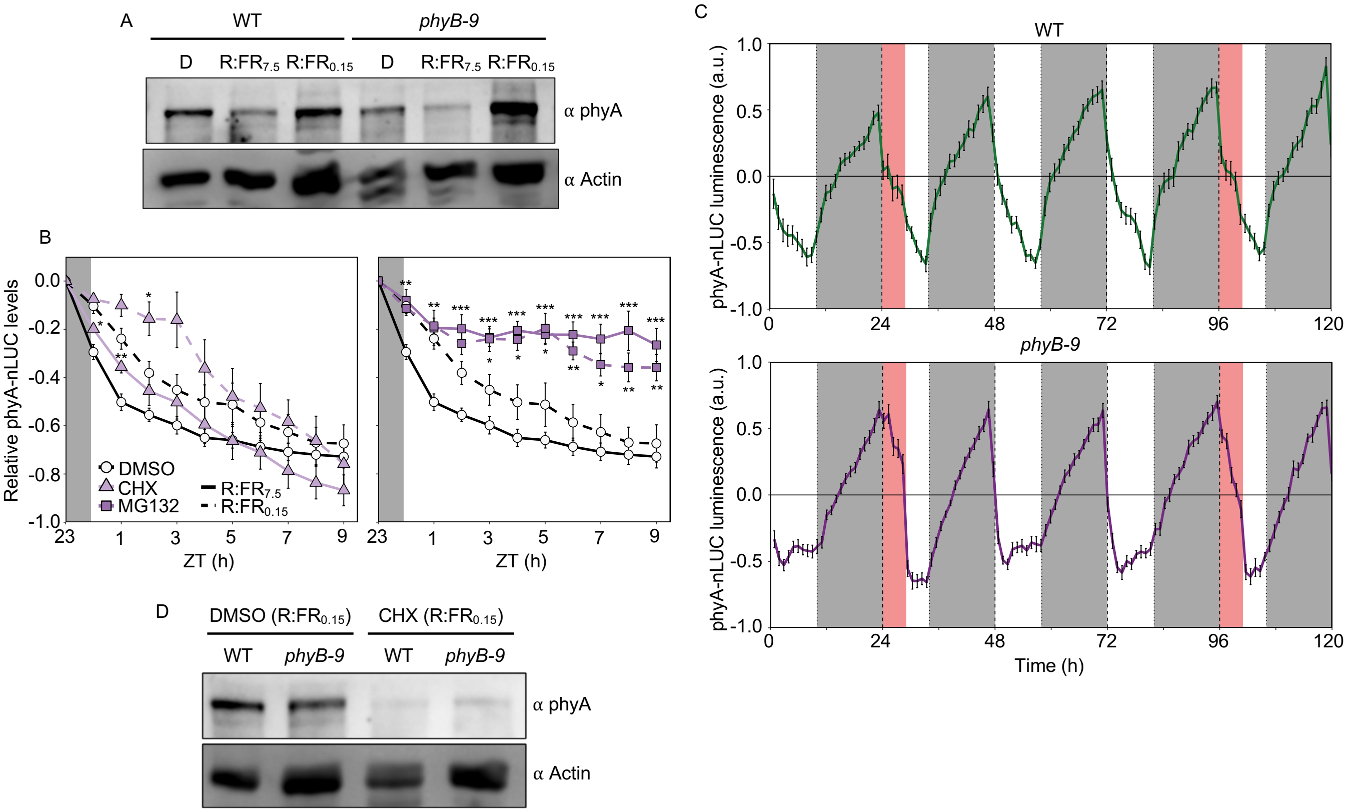

Fig. S9. Change in phyA protein during low R:FR periods is enhanced in a *phyB-9* background. (*A*) Native phyA protein levels in WT or *phyB-9,* shown in a representative western blot (supporting Fig. 1 *E*). Seedlings were sampled just prior to dark-light transition (D), or at midday (ZT5) in R:FR$_{7.5}$ or R:FR$_{0.15}$. (*B*) Hourly abundance changes in phyA-nLUC expressed in a *phyB-9* background in R:FR$_{7.5}$ (solid lines; replotted from Fig. 1 *G*) and R:FR$_{0.15}$ (dashed lines) when treated with 50 µM MG132 to block proteasomal degradation or 200 µM CHX to block translation; DMSO alone was used as a control with the same DMSO data shown in both panels. Relative phyA-nLUC levels following the dark-light transition (ZT0) were calculated with respect to the point of pre-dawn inhibitor application (ZT23). Relative change ([value at ZTX - value at ZT23] / value at ZT23) calculated for each seedling, then averaged. Asterisks indicate significant differences between R:FR$_{7.5}$ and R:FR$_{0.15}$ within the same inhibitor treatments at each time point (*$P < 0.05$; **$P < 0.01$; ***$P < 0.001$), calculated using a Student's t-test. (*C*) PhyA-nLUC bioluminescence across diurnal cycles in WT (green line, top panel) or a *phyB-9* (purple line, bottom panel) background. Two morning (ZT0-5) 5 h periods of R:FR$_{0.15}$ were provided: one from 24-29 h and one from 96-101 h. Traces show mean signal produced by $n \geq 8$ plants, measured at 1 h intervals; error bars show ± SEM. Background colours of each panel correspond to R:FR$_{7.5}$ (white), R:FR$_{0.15}$ (red) and night (grey) periods. (*D*) Native phyA protein in WT or *phyB-9* seedlings at ZT5 (R:FR$_{0.15}$) when treated with CHX or a control (DMSO), shown in a representative western blot (supporting Fig. 1 *J*). All experiments were conducted in a background WL of 15 µmol m^{-2} s^{-1}. Experiments were repeated a minimum of three times.

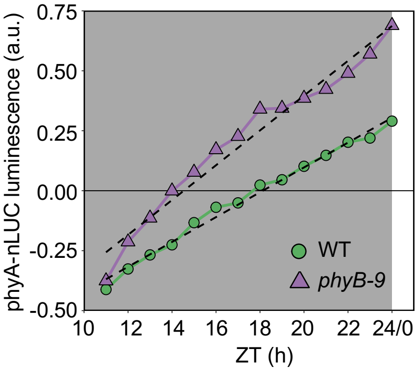

Fig. S10. Loss of phyB accelerates night time accumulation of phyA-nLUC. Night-time (ZT10-24) bioluminescence of phyA-nLUC (arbitrary units, a.u.) from entrained WT (green circles) and *phyB-9* (purple triangles) seedlings between. Fitted regression lines for WT (R^2 = 0.99, p < 0.001) and *phyB-9* backgrounds (R^2 = 0.97, p < 0.001) had the following equations: WT, y = 0.052(x) - 0.939; *phyB-9*, y = 0.073(x) - 1.055. Traces represent mean values ± SEM (n ≥ 10). Background colours of panels correspond to day (white) and night (grey) periods. Seedlings were entrained in 15 µmol m^{-2} s^{-1} WL (R:$FR_{7.5}$; 10L:14D) for 10 days prior to nLUC signal quantification. Experiments were repeated 3 times.

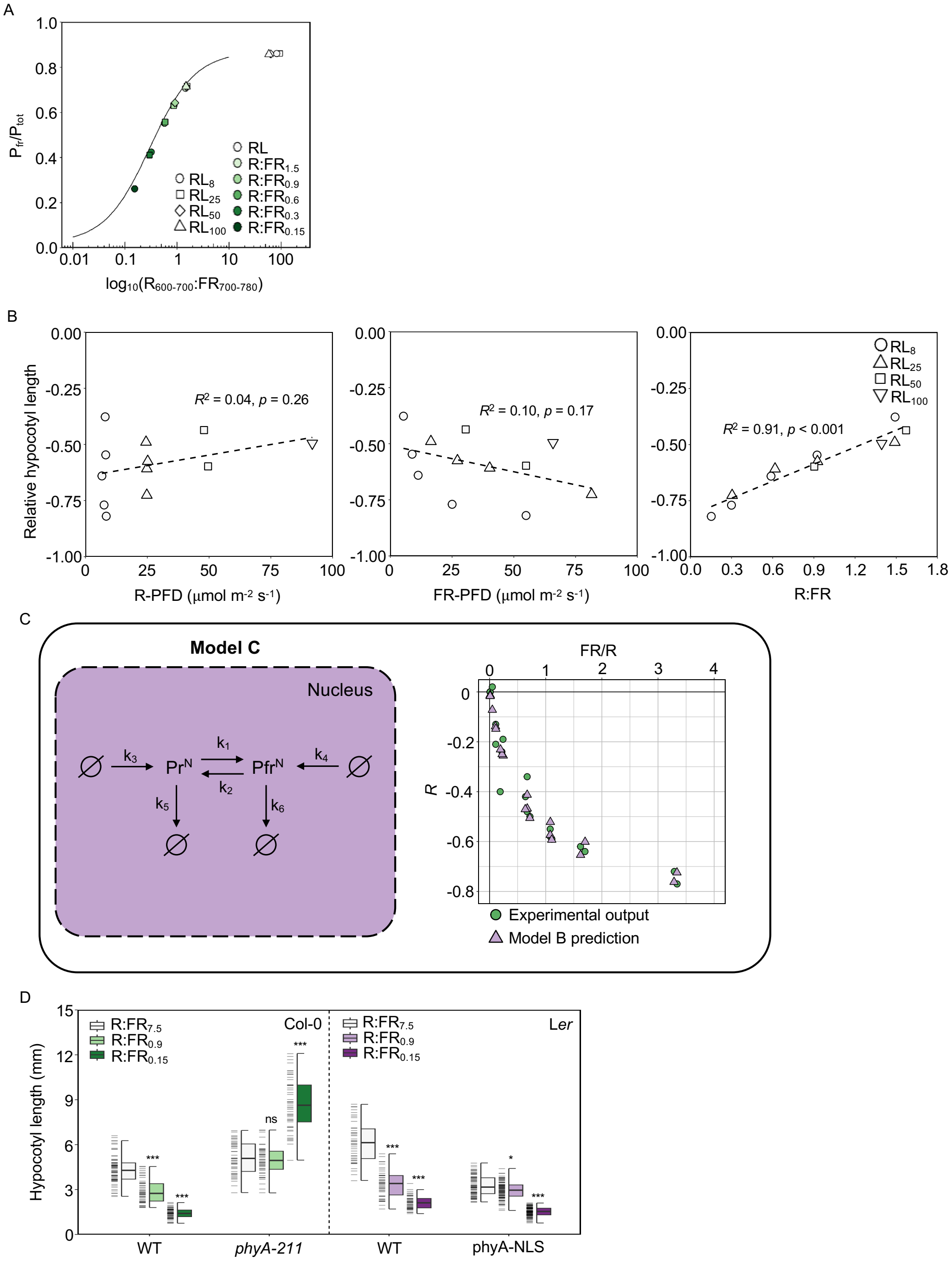

Fig. S11. PhyA growth suppression correlates with external R:FR ratios. (*A*) Predicted Pfr/Ptot ratios at corresponding R:FR (600-700 nm:700-780 nm) treatments used in experimental data shown in Fig. 3 *A*. R:FR ratios are plotted on a logarithmic scale. These predicted measurements correlate with the predicted phytochrome photoequilibrium from Mancinelli (1994), shown as the solid line, which was obtained using the R package photobiologyPlants (v0.4.1). (*B*) Relative change in WT hypocotyl length (raw data shown in Fig. 3 *A*) under different R:FR ratios, calculated with respect to the average length of seedlings in the corresponding red light intensities (i.e. relative change = [length in R+FR - average length in R]/average length in R). Linear regression analysis reveals a significant correlation between hypocotyl suppression and R:FR but not with R photon flux density (R-PFD) or FR-PFD. (*C*) Simplified version of Model B shown in Fig 3 *B*, which explains

phyA growth suppression under R:FR conditions to a similar degree of accuracy. Pr^N and Pfr^N show nuclear phyA molecules. Ø represents synthesis and degradation sources, and $k_{1\text{-}6}$ reaction parameters The plot to the right of the models shows the predicted (triangles) and actual (green circles, obtained from experimental work) relative hypocotyl length values (*R* = [value in R+FR light – average value in red light] / average value in red light), plotted against a function of far-red to red light (FR/R). (*D*) Hypocotyl lengths of 6-day-old *phyA-211* and phyA-NLS (*pPHYA::PHYA-sGFP-NLS*) seedlings grown in R:FR ratios (7.5, 0.9 or 0.15; 10hL:14hD, PAR = 15 μmol m^{-2} s^{-1}) compared to their respective wild-type backgrounds (Col-0 and L*er*, indicated in the top right of each panel). Each box plot shows as follows: median (centre line); upper and lower quartiles (box limits); 1.5 x interquartile range (whiskers); individual data points (dashes on the left of boxes, $n \geq 25$). Asterisks indicate significant differences from $R:FR_{7.5}$ controls (*$P < 0.05$; **$P < 0.01$; ***$P < 0.001$) calculated using a Wilcoxon signed-rank test.

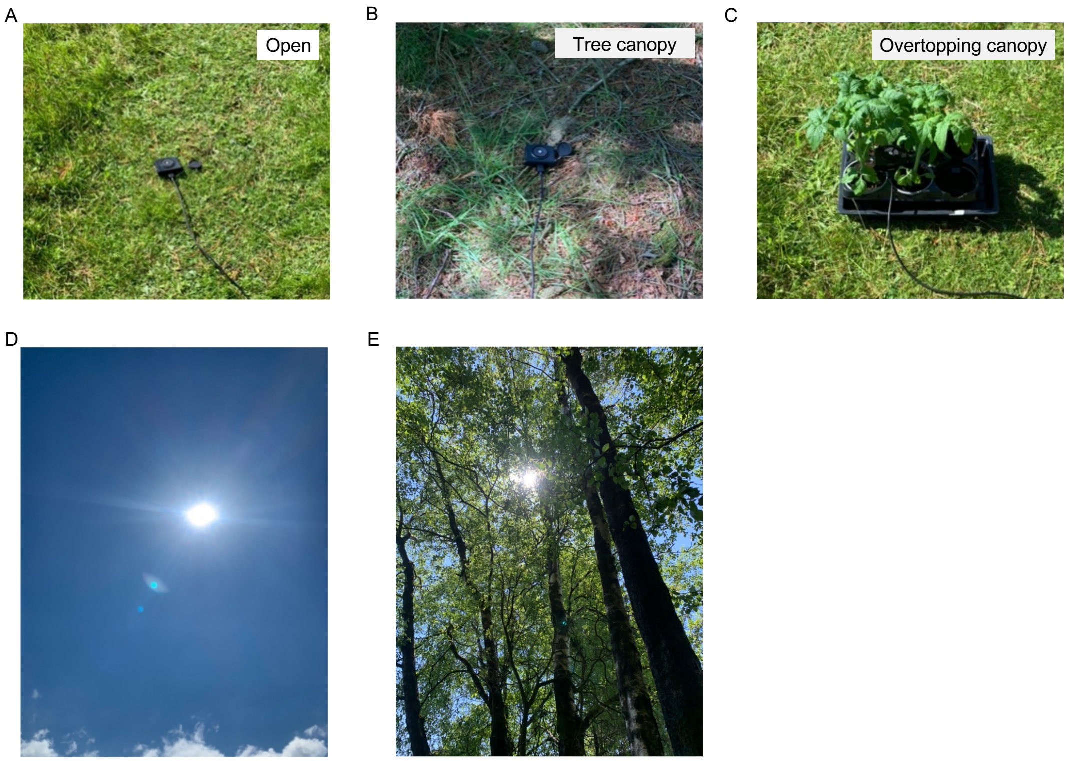

Fig. S12. Images of the environments used for spectral data collection. The open site had no overhead canopy cover (*A* and *D*) causing shading of the spectrometer at any point during the day. Two canopy shade environments consisted of a natural heterogenous woodland ('tree canopy'; *B* and *E*), which existed ~ 20 m from the 'open' measurement site or the addition of four ~ 30 cm tall *Solanum lycopersicum* plants to create an 'overtopping canopy' closer to ground-level (*C*). Images were taken ~ 13:00 on 4th June 2022, near Leadburn, Scotland (UK) (55° 46' 30.0" N, 3° 13' 13.3" W).

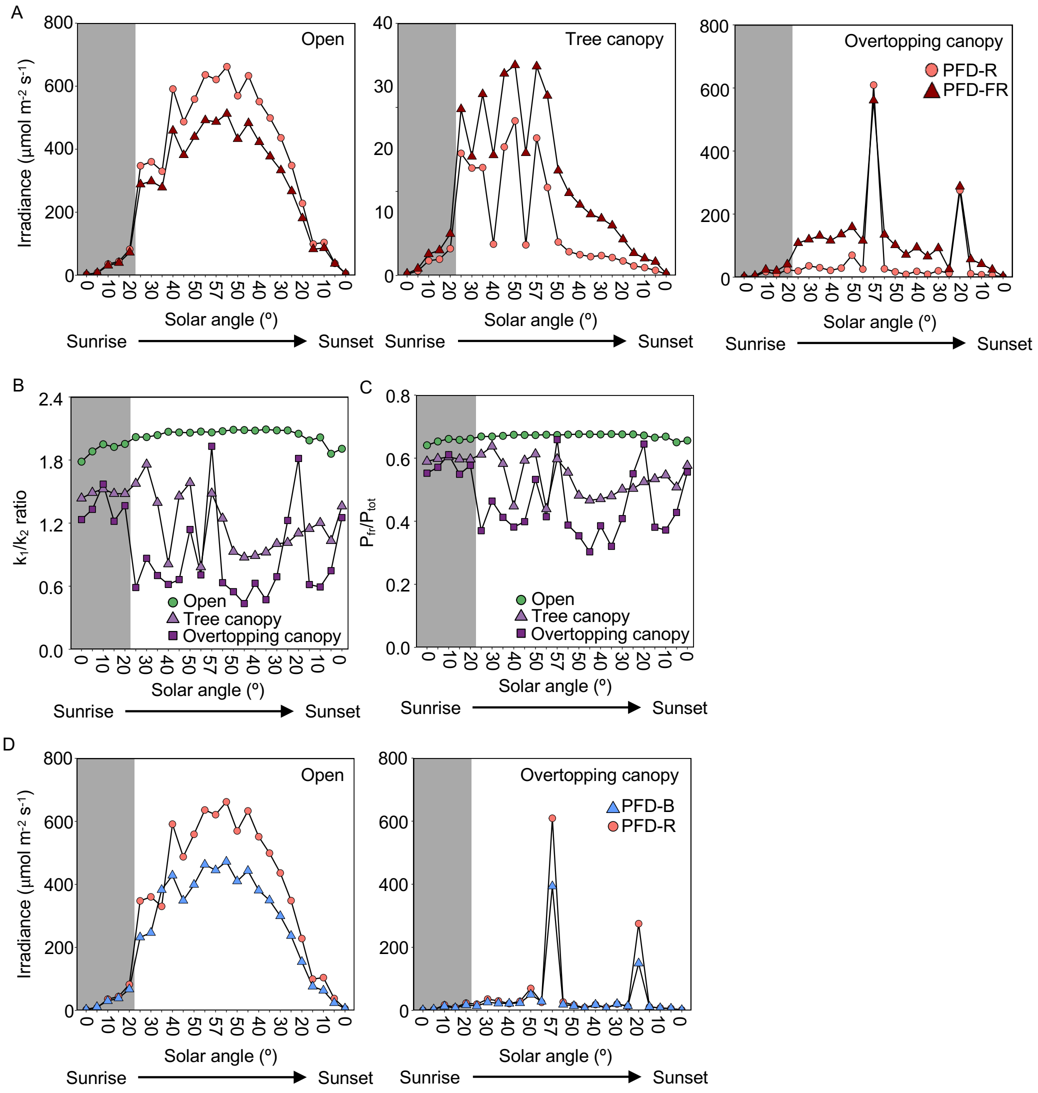

Fig. S13. Variation in the spectral make up of open and canopy shade environments and their impacts on the phytochrome equilibrium. (*A*) Comparison of the photon flux density (PFD)-red (R; 600-700 nm) vs PFD-far-red (FR; 700-780 nm) irradiances from sunrise to sunset in open and canopy shade environments. (*B*) Predicted ratios between k_1 (Pr → Pfr) and k_2 (Pfr → Pr) transition rate in natural open and canopy shade environments. Ratios were calculated using spectral irradiances (380-780 nm) integrated with the Pr and Pfr photoconversion spectra published in Mancinelli (1994). (*C*) Predicted Pfr/Ptot ratios from spectral data plotted against the $\log_{10}$ R:FR (600-700 nm:700-780 nm). Solid line indicates the predicted phytochrome photoequilibrium from Mancinelli (1994) calculated in monochromatic R and FR light, which was obtained using the R packaged photobiologyPlants (v0.4.1). (*D*) PFD-blue (B; 400-500 nm) vs PFD-R from the open and overtopping canopy environments; tree canopy shade data are plotted in Fig. 4 *E*. Dark grey panel background indicates cloudy weather conditions.

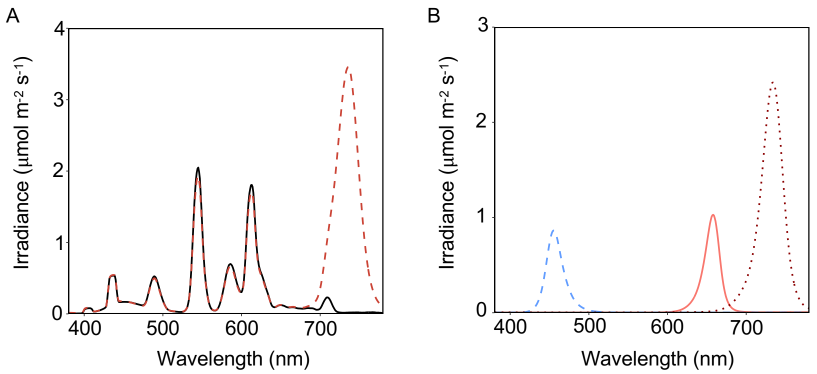

Fig. S14. Spectral outputs of cabinets used in experimental work. (*A*) Spectra of light in Percival I30-BL incubators fitted with Luxline Plus F18W/840 fluorescent tubes. Solid black line shows emission of fluorescent tubes only (i.e. WL or high R:FR); dashed red line shows the effects of additional FR supplementation using OLSON 150 6+ Series FR LED strips to generate a low R:FR (0.3) condition. (*B*) Spectral outputs of Phillips GreenPower 'blue' (dashed blue line), 'deep red' (solid red line) and 'far-red' (dotted dark red line) LEDs used to generate combinations of B ± R ± FR combinations.

Table S1. R:FR calculations across commonly used waveband definitions, along with corresponding phytochrome transition rate and Pfr/Ptot predictions for conditions used in Fig. 2 *A*.

		R:FR measurements				
Red intensity (μmol m^{-2} s^{-1})	Treatment	600-700nm: 700-780nm	640-700nm: 700-760nm	640-670nm: 720-750nm	k_1/k_2 transition ratio	Pfr/Ptot
8						
	RL	82.35	97.62	191.10	6.20	**0.86**
	R:FR$_{1.5}$	1.46	1.32	1.71	2.41	**0.71**
	R:FR$_{0.9}$	0.88	0.80	0.95	1.71	**0.63**
	R:FR$_{0.6}$	0.58	0.53	0.59	1.23	**0.55**
	R:FR$_{0.3}$	0.32	0.30	0.30	0.74	**0.42**
	R:FR$_{0.15}$	0.15	0.15	0.11	0.35	**0.26**
25						
	RL	94.21	110.70	215.69	6.20	**0.86**
	R:FR$_{1.5}$	1.56	1.41	1.72	2.53	**0.72**
	R:FR$_{0.9}$	0.87	0.79	0.92	1.70	**0.63**
	R:FR$_{0.6}$	0.59	0.54	0.60	1.26	**0.56**
	R:FR$_{0.3}$	0.30	0.28	0.27	0.70	**0.41**
50						
	RL	63.53	76.25	144.13	6.08	**0.86**
	R:FR$_{1.5}$	1.51	1.38	1.62	2.50	**0.71**
	R:FR$_{0.9}$	0.92	0.84	0.95	1.80	**0.64**
100						

RL	58.51	69.76	128.94	6.01	**0.86**
R:$FR_{1.5}$	1.50	1.39	1.55	2.50	**0.71**

Note: k_1/k_2 transition and Pfr/Ptot ratios were predicted according to Pr/Pfr conversion spectra of oat phyA, published by Mancinelli (1994). Calculations and scripts were obtained from Dr. Johanna Kramer.

Table S2. Summary of spectral data collected on 04.06.2022.

Environment	Solar angle	Approx. measurement time	Cloud cover	PFD measurements (μmols m^{-2} s^{-1})						R:FR
				PAR (400-700 nm)	UV (380-400nm)	Blue (400-500nm)	Green (500-600nm)	Red (600-700nm)	Far-red (700-780nm)	
Open										
	0°	04:30	Cloudy	7.19	0.26	2.73	2.23	2.28	2.19	1.04
	5°	05:21	Cloudy	28.40	1.07	10.70	9.50	8.39	7.41	1.13
	10°	06:03	Cloudy	98.84	2.76	29.91	34.07	35.53	30.94	1.15
	15°	06:43	Cloudy	125.76	3.70	38.61	44.13	43.90	39.42	1.11
	20°	07:19	Cloudy	229.00	6.15	67.01	81.29	82.30	72.26	1.14
	25°	07:54	Clear	892.77	18.90	231.94	319.51	347.57	288.51	1.20
	30°	08:30	Clear	934.98	20.44	246.28	335.06	360.20	298.18	1.21
	35°	09:06	Clear	1415.81	284.67	382.55	407.82	330.12	278.77	1.18
	40°	09:44	Clear	1571.81	37.03	428.28	563.39	591.21	458.96	1.29
	45°	10:24	Clear	1289.08	29.75	348.73	462.01	487.40	381.65	1.28
	50°	11:07	Clear	1476.71	34.11	398.84	529.37	558.89	439.23	1.27
	55°	12:06	Clear	1694.14	40.20	462.85	607.20	636.01	492.12	1.29
	57°	13:11	Clear	1642.81	38.53	444.93	588.21	621.22	487.02	1.28
	55°	14:00	Clear	1748.53	40.50	472.14	626.73	661.96	512.35	1.29
	50°	15:06	Clear	1509.21	35.32	410.03	540.13	569.66	432.70	1.32
	45°	15:55	Clear	1658.79	37.11	443.03	594.31	633.12	482.85	1.31
	40°	16:33	Clear	1437.19	31.50	380.87	515.22	551.22	422.84	1.30
	35°	17:09	Clear	1305.97	29.24	349.44	466.65	499.06	377.17	1.32

	30°	17:47	Clear	1131.38	24.65	299.52	403.73	436.09	333.26	1.31
	25°	18:21	Clear	898.01	19.42	236.66	319.24	348.41	266.73	1.31
	20°	18:57	Clear	584.47	12.75	154.15	206.36	228.04	180.91	1.26
	15°	19:35	Clear	265.63	6.90	75.95	92.30	99.21	82.81	1.20
	10°	20:12	Clear	251.79	4.97	63.03	86.52	103.95	86.16	1.21
	5°	20:54	Clear	89.82	1.98	23.64	29.14	37.61	36.52	1.03
	0°	21:41	Clear	16.86	0.53	6.01	5.36	5.60	4.67	1.20
Tree canopy										
	0°	04:30	Cloudy	0.88	0.03	0.32	0.29	0.28	0.43	0.65
	5°	05:21	Cloudy	3.30	0.11	1.10	1.15	1.08	1.61	0.67
	10°	06:03	Cloudy	9.59	0.25	2.73	3.44	3.49	5.09	0.69
	15°	06:43	Cloudy	10.92	0.30	3.16	3.98	3.86	5.98	0.65
	20°	07:19	Cloudy	17.60	0.45	4.87	6.48	6.37	9.90	0.64
	25°	07:54	Clear	76.39	1.63	19.42	28.45	29.05	39.59	0.73
	30°	08:30	Clear	70.12	1.70	19.30	25.76	25.56	28.28	0.90
	35°	09:06	Clear	69.29	1.63	18.00	26.11	25.67	43.16	0.59
	40°	09:44	Clear	25.62	0.99	8.11	10.24	7.45	28.63	0.26
	45°	10:24	Clear	83.45	2.04	22.18	31.27	30.58	48.03	0.64
	50°	11:07	Clear	99.47	2.37	26.57	36.80	36.79	50.05	0.74
	55°	12:06	Clear	24.78	0.94	7.63	10.05	7.27	29.14	0.25
	57°	13:11	Clear	88.90	2.15	23.48	33.34	32.70	49.76	0.66
	55°	14:00	Clear	59.33	1.58	16.14	22.66	20.93	42.77	0.49
	50°	15:06	Clear	25.91	0.96	7.99	10.15	7.94	24.98	0.32
	45°	15:55	Clear	20.22	0.89	6.96	7.76	5.64	19.61	0.29
	40°	16:33	Clear	17.91	0.80	6.28	6.82	4.94	16.82	0.29

	35°	17:09	Clear	16.96	0.81	6.29	6.32	4.47	14.55	0.31
	30°	17:47	Clear	17.38	0.78	6.33	6.43	4.74	13.55	0.35
	25°	18:21	Clear	15.51	0.68	5.60	5.76	4.25	11.89	0.36
	20°	18:57	Clear	13.07	0.58	4.96	4.76	3.44	8.59	0.40
	15°	19:35	Clear	9.23	0.44	3.75	3.28	2.26	5.34	0.42
	10°	20:12	Clear	6.97	0.27	2.65	2.51	1.87	4.05	0.46
	5°	20:54	Clear	3.85	0.12	1.32	1.35	1.20	3.24	0.37
	0°	21:41	Clear	0.84	0.03	0.28	0.27	0.29	0.49	0.60

Overtopping canopy										
	0°	04:30	Cloudy	2.26	0.07	0.78	0.78	0.71	1.40	0.51
	5°	05:21	Cloudy	9.15	0.34	3.29	3.29	2.63	4.71	0.56
	10°	06:03	Cloudy	46.86	1.14	12.94	16.91	17.33	23.69	0.73
	15°	06:43	Cloudy	27.39	0.72	7.56	10.48	9.54	20.13	0.47
	20°	07:19	Cloudy	64.12	1.56	17.24	24.23	23.10	40.57	0.57
	25°	07:54	Clear	60.56	1.55	13.99	27.25	19.72	108.41	0.18
	30°	08:30	Clear	105.69	2.65	25.79	44.80	35.81	119.73	0.30
	35°	09:06	Clear	94.54	2.58	22.37	42.82	29.99	131.25	0.23
	40°	09:44	Clear	76.71	2.82	21.91	33.71	21.59	116.00	0.19
	45°	10:24	Clear	95.81	2.87	23.53	44.17	28.75	135.86	0.21
	50°	11:07	Clear	197.07	4.91	50.08	78.67	69.67	158.70	0.44
	55°	12:06	Clear	92.63	3.61	28.19	39.56	25.51	115.62	0.22
	57°	13:11	Clear	1560.71	31.50	393.69	568.70	609.32	560.61	1.09
	55°	14:00	Clear	81.67	2.07	18.61	37.14	26.47	134.72	0.20
	50°	15:06	Clear	57.89	1.80	14.11	27.38	16.78	101.66	0.17
	45°	15:55	Clear	29.23	0.86	6.66	13.98	8.78	71.03	0.12

40°	16:33	Clear	66.68	2.45	18.18	30.44	18.49	94.15	0.20
35°	17:09	Clear	29.80	0.82	6.99	14.06	8.94	65.86	0.14
30°	17:47	Clear	71.42	2.55	20.59	31.11	20.20	91.81	0.22
25°	18:21	Clear	41.27	1.56	13.81	15.14	12.60	25.39	0.50
20°	18:57	Clear	662.26	9.99	149.24	242.64	275.02	287.51	0.96
15°	19:35	Clear	38.70	1.31	11.46	16.62	10.88	56.80	0.19
10°	20:12	Clear	25.73	0.73	7.17	11.02	7.71	41.64	0.19
5°	20:54	Clear	18.52	0.51	5.59	7.14	5.91	23.77	0.25
0°	21:41	Clear	4.04	0.11	1.28	1.36	1.43	2.70	0.53

Note: R:FR was obtained by dividing PFD-R (600-700 nm) measurements by PFD-FR (700-780 nm).

Table S3. R:FR calculations using different waveband definitions, along with corresponding phytochrome transition rate and Pfr/Ptot predictions from spectral data.

				R:FR measurements				
Environment	Solar angle	Approx. measurement time	Cloud cover	600-700nm:700-780nm	640-700nm:700-760nm	640-670nm:720-750nm	k1/k2 transition ratio	Pfr/Ptot
Open								
	0°	04:30	Cloudy	1.03	0.89	0.85	1.78	**0.64**
	5°	05:21	Cloudy	1.12	0.91	0.90	1.88	**0.65**
	10°	06:03	Cloudy	1.14	0.93	0.91	1.95	**0.66**
	15°	06:43	Cloudy	1.10	0.89	0.87	1.92	**0.66**
	20°	07:19	Cloudy	1.13	0.90	0.88	1.95	**0.66**
	25°	07:54	Clear	1.19	0.95	0.94	2.02	**0.67**
	30°	08:30	Clear	1.20	0.95	0.94	2.02	**0.67**
	35°	09:06	Clear	1.22	0.97	0.95	2.04	**0.67**
	40°	09:44	Clear	1.28	1.00	1.00	2.07	**0.67**
	45°	10:24	Clear	1.26	1.00	0.99	2.06	**0.67**
	50°	11:07	Clear	1.26	0.99	0.99	2.06	**0.67**
	55°	12:06	Clear	1.28	1.01	1.00	2.07	**0.67**
	57°	13:11	Clear	1.26	1.00	0.99	2.06	**0.67**
	55°	14:00	Clear	1.28	1.01	1.00	2.07	**0.67**
	50°	15:06	Clear	1.30	1.02	1.02	2.09	**0.68**
	45°	15:55	Clear	1.30	1.02	1.02	2.09	**0.68**

	40°	16:33	Clear	1.29	1.02	1.01	2.08	**0.68**
	35°	17:09	Clear	1.31	1.03	1.03	2.09	**0.68**
	30°	17:47	Clear	1.30	1.02	1.01	2.08	**0.68**
	25°	18:21	Clear	1.29	1.02	1.02	2.08	**0.68**
	20°	18:57	Clear	1.25	1.00	0.98	2.05	**0.67**
	15°	19:35	Clear	1.19	0.95	0.93	1.99	**0.67**
	10°	20:12	Clear	1.19	0.98	0.97	2.02	**0.67**
	5°	20:54	Clear	1.02	0.88	0.84	1.86	**0.65**
	0°	21:41	Clear	1.19	1.00	0.97	1.91	**0.66**
Tree canopy								
	0°	04:30	Cloudy	0.64	0.57	0.51	1.44	**0.59**
	5°	05:21	Cloudy	0.66	0.57	0.52	1.49	**0.60**
	10°	06:03	Cloudy	0.68	0.58	0.53	1.52	**0.60**
	15°	06:43	Cloudy	0.64	0.54	0.49	1.48	**0.60**
	20°	07:19	Cloudy	0.64	0.54	0.49	1.48	**0.60**
	25°	07:54	Clear	0.73	0.60	0.55	1.58	**0.61**
	30°	08:30	Clear	0.89	0.73	0.68	1.76	**0.64**
	35°	09:06	Clear	0.59	0.49	0.44	1.40	**0.58**
	40°	09:44	Clear	0.26	0.21	0.17	0.81	**0.45**
	45°	10:24	Clear	0.63	0.52	0.47	1.46	**0.59**
	50°	11:07	Clear	0.73	0.60	0.55	1.58	**0.61**
	55°	12:06	Clear	0.25	0.20	0.16	0.78	**0.44**
	57°	13:11	Clear	0.65	0.54	0.49	1.48	**0.60**
	55°	14:00	Clear	0.48	0.41	0.35	1.24	**0.55**
	50°	15:06	Clear	0.31	0.26	0.22	0.93	**0.48**
	45°	15:55	Clear	0.28	0.23	0.19	0.87	**0.47**

	40°	16:33	Clear	0.29	0.24	0.20	0.89	**0.47**
	35°	17:09	Clear	0.30	0.25	0.21	0.92	**0.48**
	30°	17:47	Clear	0.34	0.29	0.24	1.00	**0.50**
	25°	18:21	Clear	0.35	0.29	0.25	1.01	**0.50**
	20°	18:57	Clear	0.40	0.33	0.28	1.10	**0.52**
	15°	19:35	Clear	0.42	0.35	0.30	1.15	**0.53**
	10°	20:12	Clear	0.45	0.38	0.34	1.20	**0.55**
	5°	20:54	Clear	0.37	0.33	0.28	1.03	**0.51**
	0°	21:41	Clear	0.60	0.55	0.47	1.36	**0.58**
Overtopping canopy								
	0°	04:30	Cloudy	0.50	0.45	0.38	1.23	**0.55**
	5°	05:21	Cloudy	0.55	0.47	0.41	1.33	**0.57**
	10°	06:03	Cloudy	0.72	0.61	0.56	1.57	**0.61**
	15°	06:43	Cloudy	0.47	0.40	0.34	1.22	**0.55**
	20°	07:19	Cloudy	0.56	0.47	0.42	1.37	**0.58**
	25°	07:54	Clear	0.18	0.15	0.11	0.59	**0.37**
	30°	08:30	Clear	0.29	0.24	0.20	0.86	**0.46**
	35°	09:06	Clear	0.22	0.18	0.14	0.70	**0.41**
	40°	09:44	Clear	0.18	0.14	0.11	0.62	**0.38**
	45°	10:24	Clear	0.21	0.16	0.13	0.66	**0.40**
	50°	11:07	Clear	0.43	0.36	0.30	1.14	**0.53**
	55°	12:06	Clear	0.22	0.17	0.14	0.71	**0.41**
	57°	13:11	Clear	1.08	0.87	0.83	1.93	**0.66**
	55°	14:00	Clear	0.19	0.16	0.12	0.63	**0.39**
	50°	15:06	Clear	0.16	0.12	0.09	0.55	**0.35**

45°	15:55	Clear	0.12	0.10	0.07	0.43	**0.30**
40°	16:33	Clear	0.19	0.15	0.11	0.63	**0.39**
35°	17:09	Clear	0.13	0.11	0.08	0.47	**0.32**
30°	17:47	Clear	0.22	0.17	0.13	0.69	**0.41**
25°	18:21	Clear	0.49	0.40	0.35	1.23	**0.55**
20°	18:57	Clear	0.95	0.78	0.73	1.82	**0.64**
15°	19:35	Clear	0.19	0.15	0.12	0.62	**0.38**
10°	20:12	Clear	0.18	0.15	0.11	0.59	**0.37**
5°	20:54	Clear	0.24	0.21	0.17	0.75	**0.43**
0°	21:41	Clear	0.52	0.47	0.40	1.25	**0.56**

Note: k1/k2 transition and Pfr/Ptot ratios were predicted according to Pr/Pfr conversion spectra of oat phyA, published by Mancinelli (1994). Calculations and scripts were obtained from Dr. Johanna Kramer.

Table S4. Leaf area and dry weight data, plotted in Fig. 4 *B* as LMA.

Genotype	Treatment	Blade area (mm^2)	Blade dry weight (mg)
WT			
	R:$FR_{8.5}$	253.00^{ab}	3.71^{a}
	R:$FR_{0.3}$	218.50^{a}	2.38^{b}
phyA-211			
	R:$FR_{8.5}$	216.28^{ab}	2.96^{c}
	R:$FR_{0.3}$	202.81^{a}	2.47^{b}
phyB-9			
	R:$FR_{8.5}$	238.69^{b}	2.79^{c}
	R:$FR_{0.3}$	155.71^{c}	1.52^{d}

Note: Median values shown to 2 decimal places. Statistically indistinguishable groups for respective measurements are indicated in superscript text next to each value. Differences calculated using a Kruskal-Wallis test followed by a *post-hoc* Dunn's Test (Bonferroni correction). $n \geq 20$ for WT and *phyA-211*, but $n \geq 14$ for *phyB-9* due to reduced leaf production.

Table S5. Bolt time data from adult plant experiments.

Genotype	Treatment	Median bolt day	% plants flowered at harvest (D36)	Significance group
WT				
	$R:FR_{8.5}$	34	45.8	a
	$R:FR_{0.3}$	23	100	b
phyA-211				
	$R:FR_{8.5}$	ND	ND	---
	$R:FR_{0.3}$	27	100	c
phyB-9				
	$R:FR_{8.5}$	25	100	d
	$R:FR_{0.3}$	20.5	100	e

Note: ND = No data; no *phyA-211* plants flowered by day 36 (D36). Significance between median bolt day calculated using *post-hoc* Dunn's Test with Bonferroni corrections ($n \geq 22$).

Table S6. Spectral measurements from growth cabinets used in experimental work.

Corresponding figures	Background light	Treatment	Irradiance (μmol m^{-2} s^{-1}) PAR (400-700 nm)	B (400-500nm)	G (500-600nm)	R (600-700nm)	FR (700-780nm)	R:FR
**Fig. 1**								
	WL_{15}							
		$WL/R:FR_{7.5}$	15.68	2.47	6.55	6.74	0.88	7.64
		$R:FR_{0.15}$	14.91	2.28	5.69	7.02	50.82	0.14
**Fig. 4**								
	WL_{100}							
		$R:FR_{8.5}$	100.83	21.53	43.24	36.53	4.3	8.51
		$R:FR_{0.3}$	97.94	20.9	40.95	36.56	119.35	0.31
**Fig. 3 *A***								
	RL_8							
		RL	8.12	< 0.5	< 0.5	8.04	< 0.5	87.36
		$R:FR_{1.5}$	8.08	< 0.5	< 0.5	7.95	5.33	1.49
		$R:FR_{0.9}$	8.36	< 0.5	< 0.5	8.17	8.85	0.92
		$R:FR_{0.6}$	6.76	< 0.5	< 0.5	6.63	11.28	0.59
		$R:FR_{0.3}$	7.76	< 0.5	< 0.5	7.5	25.03	0.3
		$R:FR_{0.15}$	8.9	< 0.5	< 0.5	8.38	54.94	0.15
	RL_{25}							
		RL	26.24	< 0.5	< 0.5	26.02	< 0.5	93.33
		$R:FR_{1.5}$	24.77	< 0.5	< 0.5	24.48	16.44	1.49

		$R:FR_{0.9}$	25.72	< 0.5	< 0.5	25.17	27.18	0.93
		$R:FR_{0.6}$	25.27	< 0.5	< 0.5	24.83	40.24	0.62
		$R:FR_{0.3}$	25.68	< 0.5	< 0.5	24.8	81.48	0.3
	RL_{50}							
		RL	50.47	< 0.5	< 0.5	50	0.55	90.45
		$R:FR_{1.5}$	48.71	< 0.5	< 0.5	47.95	30.51	1.57
		$R:FR_{0.9}$	50.32	< 0.5	< 0.5	49.59	54.93	0.9
	RL_{100}							
		RL	97.99	< 0.5	0.68	97.11	1.04	93.27
		$R:FR_{1.5}$	93.26	0.52	0.84	91.77	65.85	1.4
**Fig. 3 *C***								
	RL_{25}							
		RL	24.27	< 0.5	< 0.5	23.92	< 0.5	66.67
		$R:FR_{0.3}$	26.50	< 0.5	< 0.5	25.71	78.94	0.33
**Fig. 3 *E***								
	$B_8:R_8$							
		Control	15.96	8.21	< 0.5	7.57	< 0.5	49.06
		$R:FR_{0.9}$	15.98	8.40	< 0.5	7.40	8.55	0.87
		$R:FR_{0.6}$	15.64	8.10	< 0.5	7.34	12.70	0.58
		$R:FR_{0.3}$	15.88	8.02	< 0.5	7.62	26.67	0.29
	$B_8:R_{25}$							
		Control	32.52	7.66	< 0.5	24.65	< 0.5	85.36

	$R:FR_{0.9}$	33.17	7.49	< 0.5	25.4	28.76	0.88
	$R:FR_{0.6}$	32.92	7.5	< 0.5	25.12	41.02	0.61
	$R:FR_{0.3}$	35.16	7.67	< 0.5	27.08	88.5	0.31
$B_{25}:R_{25}$							
	Control	50.86	25.42	< 0.5	25.02	< 0.5	97.01
	$R:FR_{0.9}$	51.31	25.26	0.54	25.54	28.82	0.89
	$R:FR_{0.6}$	52.79	25.44	0.59	26.78	44.52	0.6
	$R:FR_{0.3}$	51.32	25.23	0.71	25.41	88	0.29

Table S7. R:FR calculations across commonly used waveband definitions from WL cabinets supplemented with FR LED lights, along with corresponding phytochrome transition rate and Pfr/Ptot predictions for data in Fig. 1 *C*, *H* and *I*, and Fig. 4 *A*-*C*.

		R:FR measurements				
PAR (μmols m^{-2} s^{-1})	Treatment	600-700nm: 700-780nm	640-700nm: 700-760nm	640-670nm: 720-750nm	k_1/k_2 transition ratio	Pfr/Ptot
15						
	$R:FR_{7.5}$	7.76	1.27	4.63	4.02	**0.80**
	$R:FR_{0.15}$	0.15	0.04	0.02	0.32	**0.24**
100						
	$R:FR_{8.5}$	8.81	1.36	5.03	3.97	**0.80**
	$R:FR_{0.3}$	0.30	0.06	0.04	0.55	**0.35**

Note: k1/k2 transition and Pfr/Ptot ratios were predicted according to Pr/Pfr conversion spectra of oat phyA, published by Mancinelli (1994). Calculations and scripts were obtained from Dr. Johanna Kramer.

Table S8. Primers used for phyA-nLUC cloning.

Gene part	Primer Sequence (5' - 3')
PHYAp FW	TCACTATGGCGGCCCCTCGATGCAACATGGGCCATGACTA
PHYAp RV	TCGGCCTAGAGCCTGACATTTTTTTTCCTGACACAGAGACA
PHYA CDS FW part 1	GTCTCTGTGTCAGGAAAAAAAAATGTCAGGCTCTAGGCCGA
PHYA CDS RV part 1	ATCCATCTCATAGTCCTTCCAAGGTAAACTCCTTGTCTTGAC
PHYA CDS FW part 2	CAAGACAAGGAGTTTACCTTGGAAGGACTATGAGATGGATGC
PHYA CDS RV part 2	TCTTCGAGTGTGAAGACCATCTTGTTTGCTGCAGCGAGTT
NL3F10H FW	AACTCGCTGCAGCAAACAAGATGGTCTTCACACTCGAAGA
NL3F10H RV	CGATCGGGGAAATTCGAGCTTCAGTGATGGTGATGGTGAT

SI references

1. A. L. Mancinelli, "The physiology of phytochrome action" in *Photomorphogenesis in Plants*, R.E. Kendrick, G.H.M. Kronenberg, Eds. (Springer, 1994), pp. 211–269.
2. U. Urquiza-Garcia, A. J. Millar, Expanding the bioluminescent reporter toolkit for plant science with NanoLUC. *Plant Methods*. 15, 68 (2019).
3. S. Nakamura, *et al.*, Gateway binary vectors with the bialaphos resistance gene, bar, as a selection marker for plant transformation. *Biosci*. *Biotechnol*. *Biochem*. 74, 1315-1319 (2010).
4. K. Wang, *Agrobacterium Protocols*. 2nd Ed. Vol. 1 (Humana Press, 2006).
5. X. Zhang, R. Henriques, S. S. Lin, Q. W. Niu, N. H. Chua, Agrobacterium-mediated transformation of Arabidopsis thaliana using the floral dip method. *Nat. Protoc*. 1, 641-646 (2006).
6. B. P. Ingalls, *Mathematical modeling in systems biology: an introduction.* (MIT press, 2013).
7. T. Zieliński, J. Hay, A.J. Millar, "Period Estimation and Rhythm Detection in Timeseries Data Using BioDare2, the Free, Online, Community Resource" in *Plant Circadian Networks*, Methods in Molecular Biology., D. Staiger, S. Davis, A.M. Davis, Eds. (Springer, 2021) pp. 15-32.
